# Machine learning-assisted high-content imaging analysis of 3D MCF7 microtissues for estrogenic effect prediction

**DOI:** 10.1038/s41598-024-53323-6

**Published:** 2024-02-06

**Authors:** Hui Li, Haitham Seada, Samantha Madnick, He Zhao, Zhaozeng Chen, Fengcheng Li, Feng Zhu, Susan Hall, Kim Boekelheide

**Affiliations:** 1 College of Pharmaceutical Sciences, Center for Drug Safety Evaluation and Research of Zhejiang University, Zhejiang University, 866 Yuhangtang Rd, Hangzhou, 310058 China; 2 Department of Pathology and Laboratory Medicine, Brown University, 70 Ship Street, Providence, RI 02903 USA; 3 College of Pharmaceutical Sciences, Zhejiang University, Hangzhou, China

**Keywords:** Biological techniques, Computational biology and bioinformatics, Environmental sciences

## Abstract

Endocrine-disrupting chemicals (EDCs) pose a significant threat to human well-being and the ecosystem. However, in managing the many thousands of uncharacterized chemical entities, the high-throughput screening of EDCs using relevant biological endpoints remains challenging. Three-dimensional (3D) culture technology enables the development of more physiologically relevant systems in more realistic biochemical microenvironments. The high-content and quantitative imaging techniques enable quantifying endpoints associated with cell morphology, cell–cell interaction, and microtissue organization. In the present study, 3D microtissues formed by MCF-7 breast cancer cells were exposed to the model EDCs estradiol (E2) and propyl pyrazole triol (PPT). A 3D imaging and image analysis pipeline was established to extract quantitative image features from estrogen-exposed microtissues. Moreover, a machine-learning classification model was built using estrogenic-associated differential imaging features. Based on 140 common differential image features found between the E2 and PPT group, the classification model predicted E2 and PPT exposure with AUC-ROC at 0.9528 and 0.9513, respectively. Deep learning-assisted analysis software was developed to characterize microtissue gland lumen formation. The fully automated tool can accurately characterize the number of identified lumens and the total luminal volume of each microtissue. Overall, the current study established an integrated approach by combining non-supervised image feature profiling and supervised luminal volume characterization, which reflected the complexity of functional ER signaling and highlighted a promising conceptual framework for estrogenic EDC risk assessment.

## Introduction

Endocrine-disrupting chemicals (EDCs) are exogenous chemicals that interfere with hormone action in the body. The current endocrine test systems have adopted in silico, in vitro, and in vivo techniques for robust assessment of endocrine activity and adverse effects in the estrogen, androgen, and thyroid hormone signaling pathways^1^. The U.S. Environmental Protection Agency Endocrine Disruptor Screening Program (EDSP), launched in 2009, utilizes a two-tiered approach, combining in vitro and in vivo tests; each tier of the EDSP requires a battery of animal-intensive and expensive tests^2,3^. There has been extensive discussion regarding the limitations of these tests, such as inadequate sensitivity, lack of mechanistically relevant endpoints, and gaps between mechanistic responses and apical adverse outcomes. From a practical standpoint, the existing test methods require a significant investment in time, cost, and use of animals^1^. Completing all EDSP Tier 1 tests for one chemical requires a minimum of 520 animals and costs between $335,100 and $964,250^3^. Developing an in vitro high-throughput screening system with physiologically relevant responses would help overcome the limitations of the current screening tests.

The morphogenesis and function of the mammary gland are orchestrated by systematic reproductive hormones, especially the estrogens, such as 17β-estradiol (E2) and its metabolites. During mammary gland development, estrogens mediate ductal formation and elongation, whereas, in the adult mammary gland, estrogens maintain both the differentiated and stem cell populations^4–6^. Moreover, estrogens stimulate cellular proliferation and thus promote breast cancer genesis and progression^7,8^. Together, these myriad estrogenic effects make mammary epithelial cell lines a favorable in vitro system for estrogen testing, and both normal and malignant mammary cell lines have been used extensively for estrogenic and anti-estrogenic chemical identification and screening^9–11^.

Although two-dimensional (2D) cell cultures have been the main in vitro chemical screening platforms, the landscape has shifted toward three-dimensional (3D) culture systems with more physiologically relevant cellular structures and realistic biochemical and biomechanical microenvironments^12,13^. In a previous study, we established a 3D culture model of MCF7 breast cancer cells in a non-adhesive agarose hydrogel platform^14^. The non-adhesive agarose hydrogels provided a scaffold-free environment with easy media changes and cell handling^15^. Compared to its 2D counterpart, the self-assembled MCF7 3D microtissues were more differentiated, indicated by apical-basal polarity and luminal space formation^16^. Furthermore, the MCF7 microtissues recapitulated the in vivo morphology of the human mammary gland and its response to an estrogenic stimulus. A follow-up investigation showed that the expression of typical estrogen-responsive genes, including progesterone receptor (PGR), PDZ-containing domain 1 (PDZK1), and amphiregulin (AREG) were up-regulated in the 3D MCF7 microtissues treated with 1 nM estradiol (E2)^14^. Moreover, a comparison between the 3D and 2D cultures with E2 exposure revealed significant differences in cell adhesion markers expression at later time points, suggesting the 3D system provides a more physiologically relevant environment for assessing cell–cell interactions^14^. Collectively, with a human tissue-like morphology and functional estrogenic pathways, the 3D MCF7 model provides a unique opportunity for developing an in vitro platform for estrogenic effect screening.

Phenotypic profiling is part of the EPA’s blueprint, widely recognized as a promising avenue to characterize biological activities^17^. Imaging profiling has become a favorable method for high throughput screening coupled with 3D cultures. Compared to biochemical or molecular endpoints, imaging provides multifaceted information regarding cell–cell interactions, microtissue structure, and function. High-content imaging systems have been widely adopted, and subsequent image analysis can quantify phenotypes for morphological profiling of a broad spectrum of cellular metrics, such as shape, staining intensity and patterns, and spatial relationships^18^. Image-based profiling can provide diverse and comprehensive biological information while generating large amounts of data requiring considerable effort in interpretation. Therefore, computational strategies are needed to facilitate data processing and enable feature identification and characterization.

Here, we describe the development of a machine learning-assisted high-content imaging analysis and morphological profiling method applied to estrogenic endocrine disrupting effect prediction using a 3D MCF7 breast cancer microtissue model. We aim to extend the application of this animal-free strategy to a broader spectrum of potential EDCs and contribute to expanding the current endocrine disruptor screening paradigm.

## Material and method

### Chemical and reagents

Cell culture media and supplements were purchased from Life Technologies, Inc (Grand Island, NY). Fetal bovine serum (FBS) was purchased from Atlanta Biologicals (Flowery Branch, GA), and dextran-coated-charcoal (DCC) stripped was purchased from Gemini Bioscience (Sacramento, CA). Estradiol (E2), ≥ 98%, was purchased from Sigma Aldrich (St. Louis, MO). Propylpyrazoletriol (PPT), ≥ 99% (HPLC), was purchased from Tocris Bioscience (Minneapolis, MN). Dimethylsulfoxide (DMSO) was purchased from Acros Organics (Fair Lawn, NJ). Agarose was purchased from Fisher Scientific (Agawam, MA). All the chemicals and reagents were obtained from commercial suppliers.

### Cell culture and chemical treatment

#### 2D cell culture

MCF-7 (ATCC No. HTB-22) human breast carcinoma cells^19^ were cultured according to the previously described protocol^20^. Briefly, the MCF7 cells were limited to use within the first 15 passages from the original purchased vial from ATCC, to control for genomic drift due to instability. The MCF7 cells were maintained in a growth medium made of phenol-red free DMEM-F12 medium containing 10% FBS, MEM nonessential amino acids, gentamicin, and 10 μg/mL insulin in a 5% CO_2_ incubator at 37 °C.

#### 3D microtissue culture

The 3D Petri Dish 12–256-small spheroids molds (Microtissues Inc., Providence, RI) was used to make non-adhesive agarose hydrogels, which were seeded with cells as previously described^20^ MCF-7 cells grown in monolayer in tissue culture flasks were trypsinized, counted and seeded into agarose hydrogels at a density of 600,000 cells/mL. MCF-7 cells were allowed to settle into recesses for 30 min before 2 mL of treatment media was added.

#### Estrogenic compound treatment

Solutions of E2, PPT, or vehicle control (DMSO) were made in treatment media made of phenol-red free DMEM-F12 medium containing 5% DCC FBS, MEM nonessential amino acids, gentamicin, and 6 ng/mL insulin. Following the seeding of MCF-7 cells into hydrogels, 2 mL of treatment media with E2, PPT, or DMSO was added. Plates were kept in a 5% CO2 incubator and cultured for up to 7 days. Treatment media was changed on day 3 and 5 of the experiment.

### RNA isolation and gene expression

MCF7 microtissues were collected from hydrogels by centrifugation, pelleted, and lysed in Tri Reagent. The total RNA was extracted according to a previous protocol^21^. Each experiment was designed with three biological replicates. For each biological replicate, seeding cells were from separate flasks, and microtissues from six hydrogels (256 microtissues/gel) were collected. RNA quantity was determined using a Nanodrop ND1000. For use in qRT-PCR, cDNA was made using the RT2 First Strand Kit (Qiagen) per the manufacturer’s instructions. qRT-PCR was performed using RT2 SYBR Green Rox qPCR Mastermix with RT2 qPCR Primer Assays (Qiagen) to determine gene expression levels of PDZ domain containing 1 (PDZK1, PPH08038E), apolipoprotein D (APOD, PPH02630A), cytochrome P450, family 1, subfamily A, polypeptide 1 (CYP1A1, PPH01271F), transforming growth factor, beta 3 (TGFB3, PPH00531F) and normalized to ribosomal protein, large P0 (RPLPO, FWD GTGTTCGACAATGGCAGCAT, REV GACACCCTCCAGGAAGCGA). Plates were run on an Applied Biosystems ViiA 7 machine using cycling conditions recommended by the manufacturer. The mean CT for the target genes and the geometric mean CT for the endogenous control (RPLP0) genes were calculated, and the mean CT for the endogenous controls was subtracted from the mean CT for each target gene within each experiment to give the Δ Mean. The _Δ_Cт Mean at each treatment (E2 or PPT) was subtracted from the control (untreated) _Δ_Cт Mean to provide the _ΔΔ_Cт for each treatment. Finally, the _ΔΔ_Cт values were raised to the power of 2 (2–_ΔΔ_Cт) to give the fold change in the target gene at each time point relative to the DMSO control.

### Imaging feature extraction

#### Cell clearing and imaging

Following treatment, microtissues were rinsed in PBS, fixed in formalin for 15 min at room temperature, rinsed in PBS twice, and then stored in PBS at 4 °C until ready to image. Before imaging, microtissues were switched to ScaleS4 containing 1:1000 Hoechst 33,342 and 1:200 rhodamine-phalloidin. ScaleS4 is composed of 40 w/v% D-(-)-sorbitol, 10 w/v% glycerol, 4 M Urea, 0.2 w/v% Triton X-100, and 15 v/v% DMSO in deionized water. After 3 h, ScaleS4 was removed. Agarose hydrogels were removed from a 12-well plate, placed on a paper towel, the extra agarose was removed from the sides, and then flipped over into a 24-well cell imaging plate (Eppendorf) containing 50 uL of fresh ScaleS4. Cell imaging was performed using an Opera Phenix™ High Content Screening System (Perkin Elmer) using a 20 × water objective (NA 1.0, HH1400421, PerkinElmer). Image stacks were taken with a 5 µm step size. A 3D image screening protocol was set up to obtain the 3D image of the MCF7 microtissues.

#### Cell counts

Based on the 3D microtissue image acquired above, the Harmony software built a cell count protocol for each microtissue’s total cell count. Briefly, channels of three views were summed, filtered to remove background noise, and bright areas above the set absolute threshold were identified via the 'find image region' method. Several positions and morphology properties (including contact area and the nearest neighbor distance) were calculated and used to filter out image artifacts. After that, nuclei were segmented within each aggregate region via the ‘find nuclei’ method, algorithm 'C'. Similarly, property calculation and filtering were performed to further select bonafide nuclear regions for counting (Supplemental_Data_1).

#### 2D image selection and feature extraction

In Harmony, the cellular region area on each image slice was measured, and the image slice with the largest cellular region area was selected as the representative 2D image of the respective microtissue. A 2D image feature extraction pipeline was built in Harmony, and the pipeline was applied to the 2D images selected above. Briefly, the pipeline identified objects, such as the image or nuclear region, and then extracted morphological features, such as area, length, roundness, and a collection of texture features. (See Supplemental_Data_2 and Supplemental_Data_3 for a detailed feature extraction pipeline and Supplemental_Data_4 for a complete list of features). A quantitative value was calculated for each feature, and a number matrix was generated and exported for further analysis.

### Image feature analysis

#### Data normalization and regrouping

A well-established analysis method designed for enhancing multi-class data normalization was adopted here to identify the optimal normalization method for the data. This method is capable of (1) normalizing the multi-class data using 168 different normalization methods/strategies, (2) evaluating the performances of every single method/strategy from multiple perspectives, and (3) comparing the performance of all these normalization methods/strategies based on a comprehensive ranking to identify superior one^22^.

Since none of the normalization method performed well in analyzing the data in the original groups, we regrouped the samples for reanalysis. For PPT, 1 nM PPT, 3 nM PPT, and 10 nM PPT were combined into the high concentration group, and the 0.1 nM PPT was referred to as the medium concentration group, respectively and the 0.01 nM PPT as low concentration group. For E2, the 0.1 nM E2 and 1 nM E2 were combined as the high concentration group, 0.0001 nM E2 and 0.001 E2 were combined as the low concentration group, and the left 0.01 nM E2 as the medium concentration group.

After regrouping the samples in the PPT group and E2 group, we then analyzed the regrouped data with the method above and successfully identified several normalization methods, which were evaluated as well performed: for the regrouped PPT data, the best normalization method is Range Scaling, and for regrouped E2 data, the best normalization method is power scaling^23^ (Supplemental_Data_5).

#### Feature selection

For multi-class data, the orthogonal partial least squares-discriminant analysis (OPLS-DA) is a commonly used strategy for identifying differential markers^24^ is therefore adopted in our studies for feature selection. The OPLS-DA was conducted by running the opls function in the ropls R package^24^. Parameters ‘orthoI’, ‘crossvalI’, and ‘predI’ of the opls function were set to ‘NA’, ‘2’, and ‘1’, respectively, which means that the number of orthogonal components will be computed and optimized based on twofold cross-validation and one predictive component. principle component analysis (PCA): The PCA was conducted via the MetaboAnalystR R package^25^.

#### Machine learning classification model

The machine learning algorithm we adopted for constructing the classification models based on our identified markers was Random Forest (RF) since our data all contains more than 2 sample groups^26^. The RF method combined several decision tree predictors and classified the samples based on the majority of votes of a series of binary questions about given features. In our study, a training set and a test set were generated by stratified sampling from the same group in a ratio of 8:2, then the training set was used to train the RF model via the randomForest function in randomForest R packages, and the parameter ntree was set to 100; finally, the test set was used to evaluate the performance of trained RF model by calculating the AUC value via the multi_roc function in multiROC R packages.

### Luminal volume acquisition and analysis

An automated system was built to perform the luminal volume acquisition and analysis using the data generated by the high-content imaging instrument. The system consists of three parts: an image processing pipeline, a deep learning pipeline, and a volumetric analysis step. The image processing pipeline first enhances the input 2D images (z-slices) then applies a sequence of image processing operations to prepare images for the classification phase. Once the images are ready, we use our deep learning classifier (that we have trained – transfer learning – using 1000 manually marked lumens) to differentiate “true” lumens from “false” ones. The volumetric analysis step re-constructs 3D lumens from the groups of nearby “true” 2D lumens identified by the classifier. Finally, the last step calculates the volume and surface area of each of these 3D re-constructed lumens. While the user interface of the system is developed using Java, the core functionality (image processing, deep learning, re-construction, and volumetric analysis) uses MATLAB R2018a image processing toolbox, transfer learning functionality, and computational geometry toolbox, respectively. The code and associated information have been archived at https://github.com/000haitham000/lumen-explorer.

### Statistical analysis

The cell count results are represented as the mean ± SD. The gene expression data are expressed as the mean ± SD value of the relative fold change. For all comparisons of the cell count and gene expression values, one-way analysis of variance (ANOVA) statistical analysis was employed with Turkey’s multiple comparisons posttest to compare among different concentrations. All analysis was carried out using GraphPad Prism software (GraphPad Software, Inc., La Jolla, California, USA).

## Results

### Estrogen stimulation alters cell counts and molecular marker gene expression of the MCF7 microtissue

**Figure 1:**
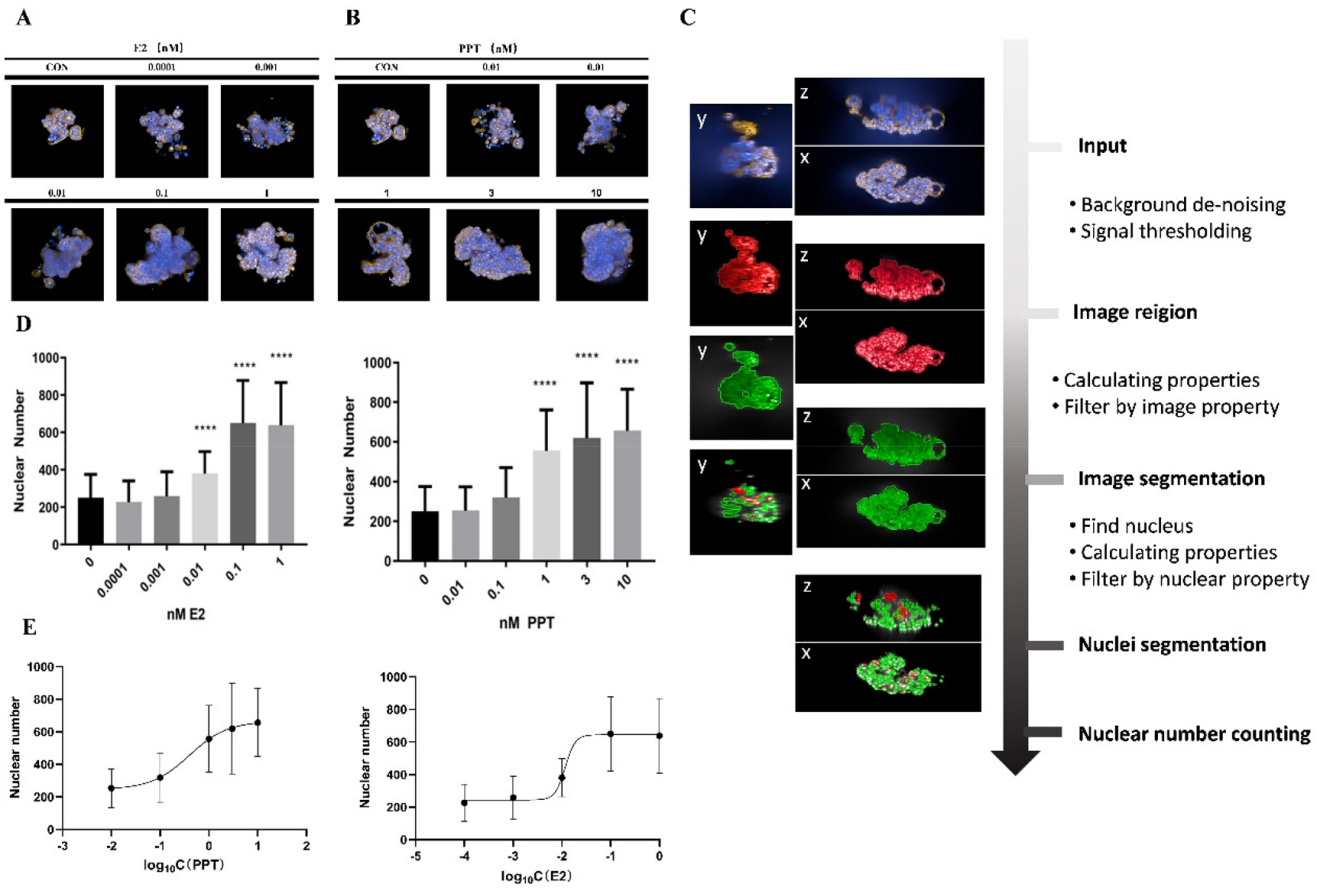
Estrogen stimulation dose-dependently increased the microtissue nuclear count. (**A**). Representative images of microtissues under E2 treatment; (**B**) Representative images of microtissues under PPT treatment; (**C**) Process of acquiring the microtissue nuclear counts; (**D**) Nuclear count microtissues under E2 and PPT treatment, (**E**) Dose–response curves of the microtissue nuclear count for E2 and PPT treatment. Data are reported as mean ± SD, n > 20, ****p < 0.0001 compared to control by one-way-ANOVA with Turkey test.

As reported in previous studies, the MCF7 cells were auto-assembled in the hydrogel microwells to form an irregular-shaped microtissue. A series of different concentrations of E2 and PPT were used for the microtissue treatment, and the 3D image reconstruction of the microtissue was obtained using a high-content imaging technique on day 7 (Fig. 1A,B). To quantify the effect of E2 and PPT, an image processing pipeline, including several steps of object identification and segmentation filtering, was established to perform a nuclear count of the microtissues (Fig. 1C, Supplemental Data 1). As shown in Fig. 1D, both E2 and PPT treatment increased the nuclear counts in a dose-dependent manner, and the EC_50_ of E2 and PPT were 0.01170 nM and 0.3986 nM, respectively. To further examine the estrogen pathway response, the gene expression of several ER downstream genes, including apolipoprotein D (APOD), cytochrome P450 1A1 (CYP1A1), transforming growth factor beta 3 (TGFB3), and PDZ containing domain 1 (PDZK1), were evaluated. The transcriptional level of APOD, CYP1A1 and TGFB3 significantly decreased compared to the untreated control from day1 to day7, whereas PDKZ1 was induced considerably over time. However, the trend of the gene expression change was non-monotonic. Compared to day1, the expression of APOD, CYP1A1, and PDKZ1 was lower on day 3 but rebounded to a level close to day 1 on day 7, suggesting a potential adaption process during the treatment time course. Under 0.1 nM E2 treatment, the fold-change of PDKZ1 was 14.41, 13.23, and 9.821 on day 1, 3, and 7, respectively, suggesting the response was maximized on early time points and slowly reduced along time. For 1 nM PPT treatment, the biggest fold change, 17.9, was observed on day3, and lowest on day 7, suggesting a relative delayed response to PPT compared to E2 (Table 1).

**Table 1:** Estrogen stimulation alters the gene expression of molecular marker in the MCF7 microtissue.

Target gene	Treatment	Day 1		Day 3		Day 7	
		Fold change	P-value	Fold change	P-value	Fold change	P-value
*APOD*	0.1 nM E2	0.3787	< 0.0001	0.1383	< 0.0001	0.04319	< 0.0001
	1 nM PPT	0.3588	< 0.0001	0.1805	< 0.0001	0.03288	< 0.0001
*PDKZ1*	0.1 nM E2	14.41	0.0423	13.23	0.1901	9.821	< 0.0001
	1 nM PPT	15.2	0.0337	17.9	0.0715	7.484	< 0.0001
*CYP1A1*	0.1 nM E2	0.3575	< 0.0001	0.07264	< 0.0001	0.2957	< 0.0001
	1 nM PPT	0.2528	< 0.0001	0.08871	< 0.0001	0.2057	< 0.0001
*TGFB3*	0.1 nM E2	0.5865	< 0.0001	0.2875	< 0.0001	0.7606	0.0154
	1 nM PPT	0.4654	< 0.0001	0.4067	< 0.0001	0.3388	< 0.0001

### Estrogen-induced MCF7 microtissue morphological alterations characterized by 2D image profiling

**Figure 2:**
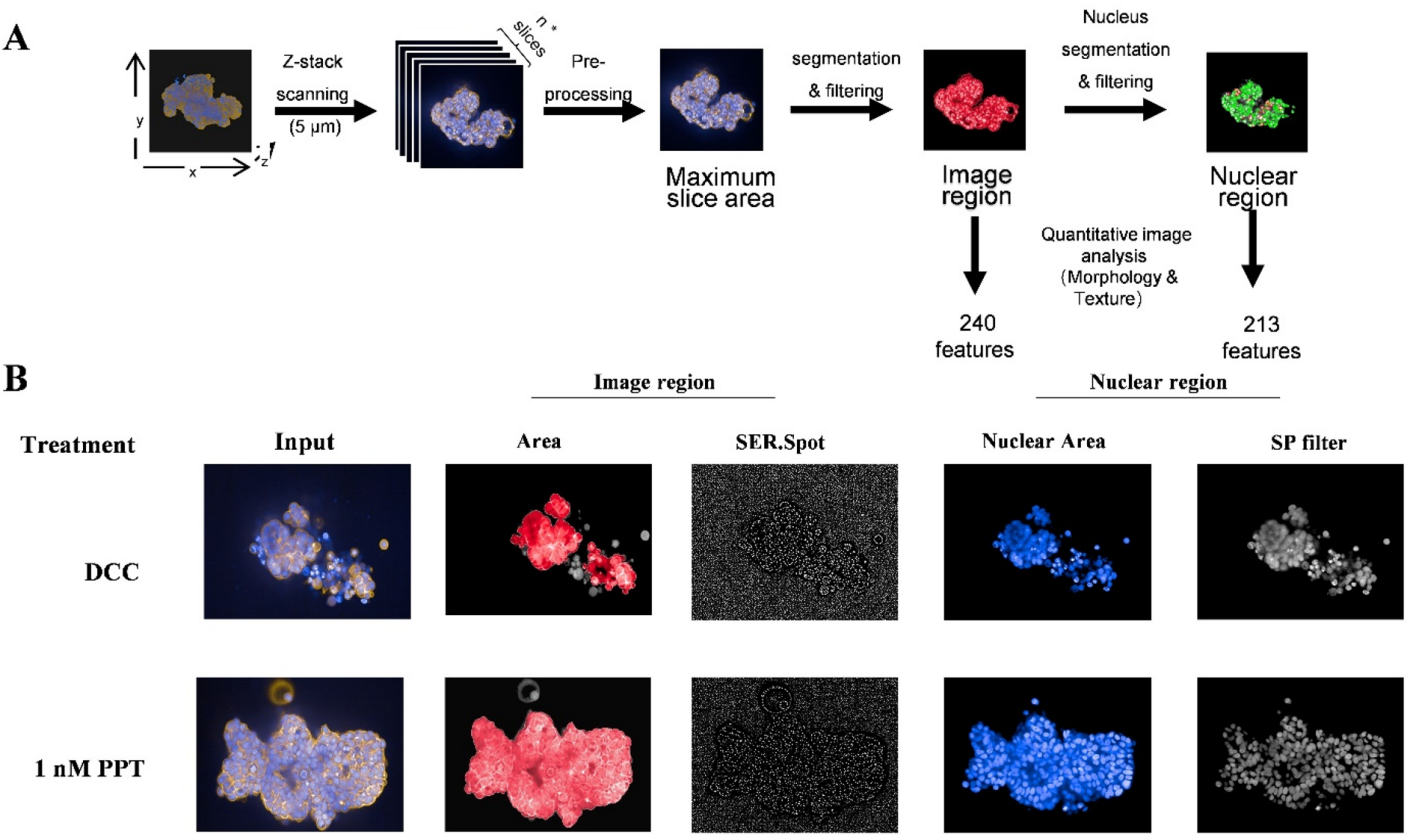
2D image slice selection and image feature acquisition. (**A**) The 2D image slice with the most extensive cellular area was selected and used as the input image for a 2D image feature extraction and quantification pipeline, in which 240 image region features and 213 nuclear region features were identified and quantified. (**B**) A comparison of the representative image features output from the 2D image analysis between control and 1 nM PPT treatment.

The 3D microtissue was imaged by Z-stack scanning every 5 μm. All 2D image slices were scanned to calculate each slice's cellular area, and the image slice with the largest cellular area was selected as the input image for a 2D image feature extraction and quantification pipeline. Through this process, the image features were gathered from two major categories, the entire image region, and the nuclear region. A total of 240 image region features and 213 nuclear region features were identified, and the quantitative values were acquired by the Harmony software (Fig. 2A). (Please refer to Supplemental_Data_4 for the complete feature lists). The representative image and nuclear region features were selected and were shown treated by DCC or 1 nM PPT, with their quantitative scores presented in (Fig. 2B).

**Figure 3:**
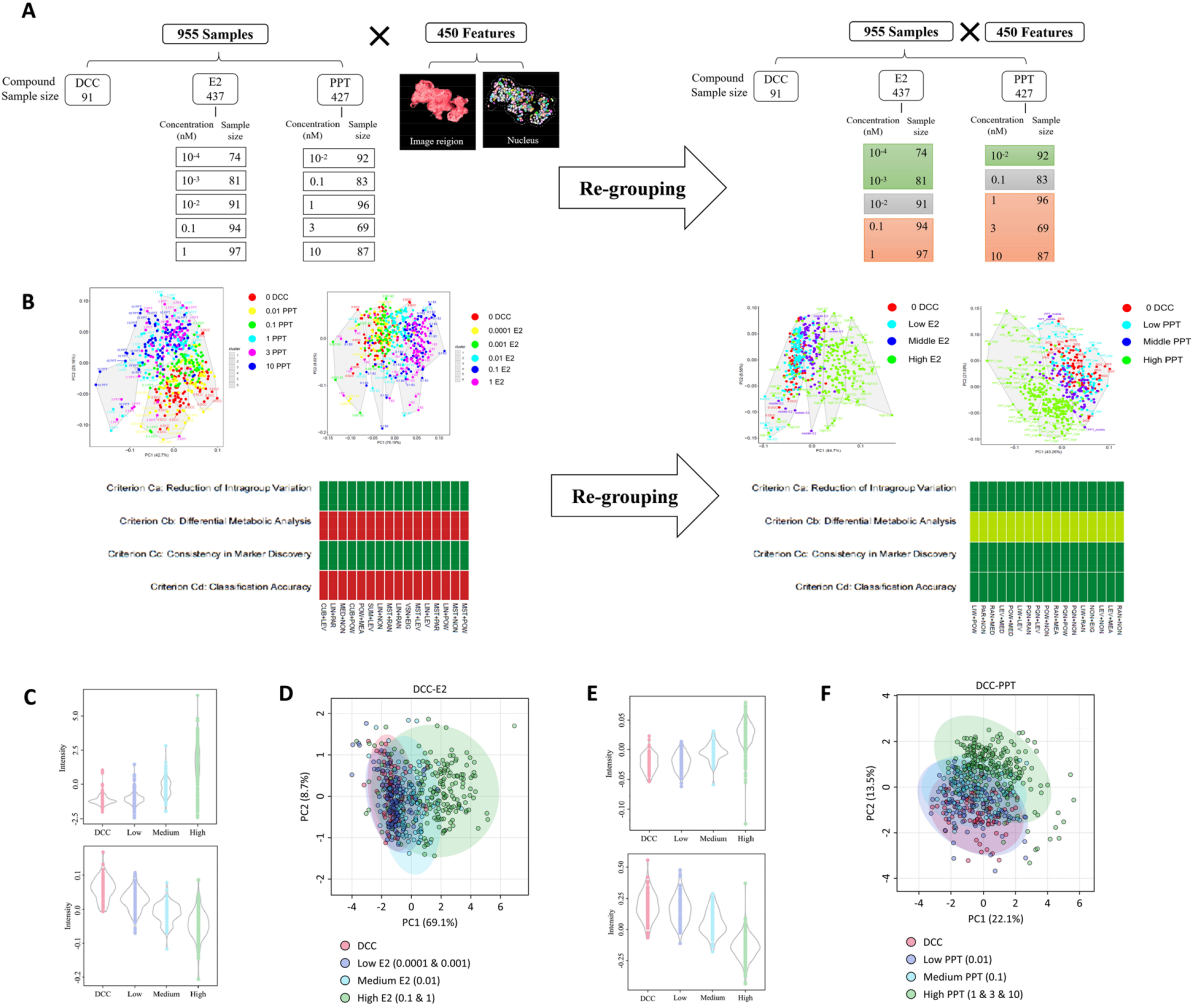
2D image profiling and differential image feature analysis. (**A**) Data matrix structure of the original and re-grouped setting of E2 and PPT concentration. The original data went through an optimal normalization selection process, and the data were normalized using the top-ranking method and used for the PCA plot. (**B**) After re-grouping, multiple normalization methods achieved minimal performance, and the top-ranking method was used to normalize the data for the PCA plot. (**C**) Violin plot of the full two ranking differential image features in response to E2 treatment. (**D**) PCA analysis for regrouped E2 dataset using selected differential image features. (**E**)Violin plot of the top two ranking differential image features in response to PPT treatment. (**F**) PCA analysis for regrouped PPT dataset using selected differential image features.

The image features describing object orientations were omitted from the original feature collection, which left 450 image features for further analysis. To identify the best normalization method for each multi-class image dataset, 168 normalization methods were evaluated for the original datasets. However, none of these methods performed well (Fig. 3A and B). Therefore, we normalized each data group using the top-ranking method and performed a PCA analysis of the normalized data. The PCA analysis showed a significant overlap of the adjacent concentration. The data were regrouped according to the initial PCA analysis to overcome the poor separation. For E2, the 0.1 nM and 1 nM E2 were combined as the high concentration group, 0.0001 nM and 0.001 E2 were combined as the low concentration group, and 0.01 nM E2 was the medium concentration group. For PPT, 1 nM PPT, 3 nM PPT, and 10 nM PPT were incorporated into the high-concentration group. The 0.1 nM PPT was referred to as the medium concentration group, and the 0.01 nM PPT as the low concentration group (Fig. 3A). After regrouping, the optimal normalization methods were re-evaluated. Several normalization methods were above the evaluation threshold, and the optimal normalization method for regrouped E2 and PPT are the Power Scaling and the Range Scaling, respectively^23^.

After regrouping, the OPLS-DA analysis was applied to identify the differential image features. The analysis identified 182 and 155 differential features for E2 and PPT, respectively. (Please refer to Supplemental_Data_6 and Supplemental_Data_7 for the complete lists of differential image features.) The violin plots for the top 2 differential image features were shown in Fig. 3C and E. For E2, the two features offered here both change in a dose-dependent manner but in different directions, and so did for PPT (Fig. 3C and E). The violin plot for the Top 10 differential features can be found in Supplemental_Data_8 and Supplemental_Data_9. Furthermore, the PCA analysis was performed as previously described. A total of 33 and 42 principal components were obtained for E2 and PPT treatment, respectively, and the first two principal components together explain 77.8% and 35.6% of the total variance, respectively (Fig. 3D and F). The PCA plots revealed clear and dose-dependent clustering patterns of each dose level (please refer to Supplemental_Data_10 for detailed PCA analysis results).

### Estrogen response prediction using the machine learning classification model

**Figure 4:**
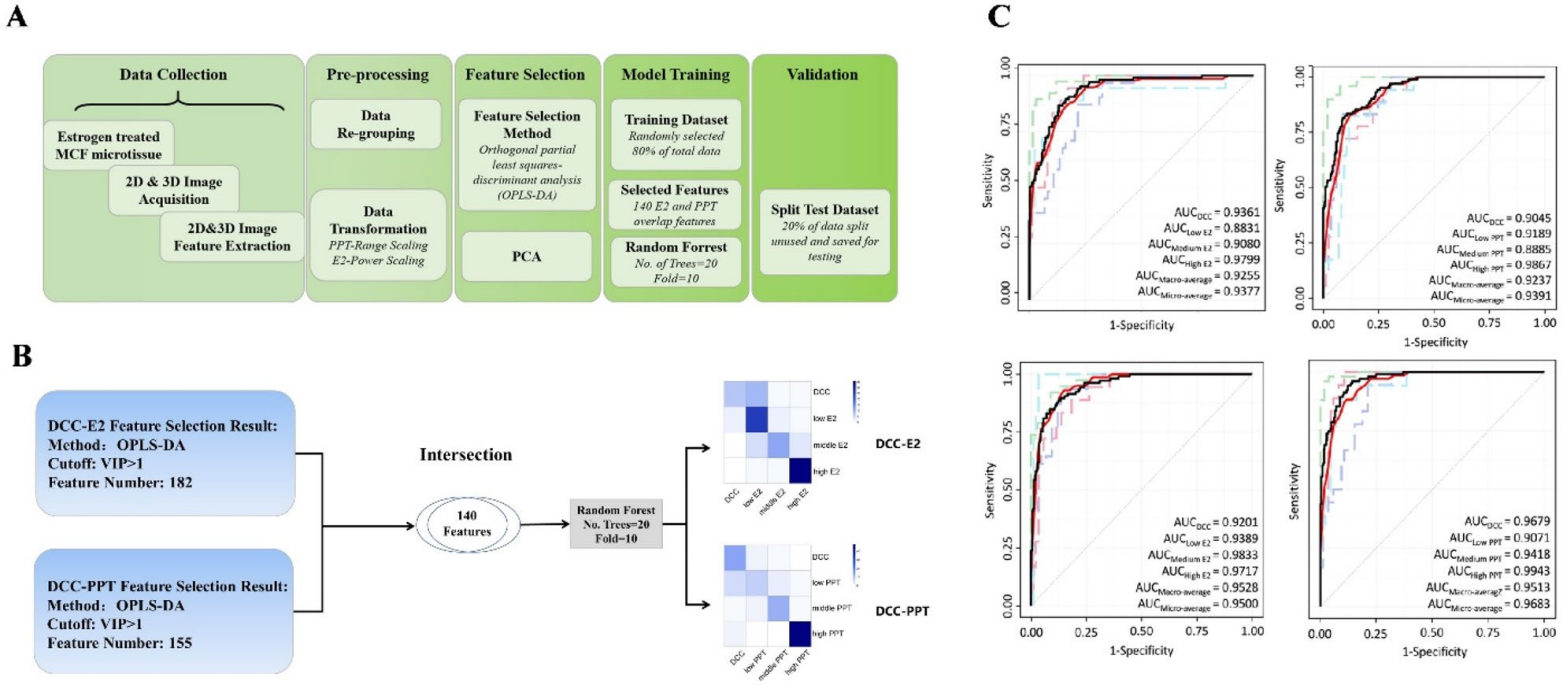
(**A**) Flow chart of prediction model building; (**B**) The data of the 140 overlapping differential features between E2 and PPT were used in the establishment of the machine learning classification model using the random forest method; (**C**) Receiver operating characteristic (ROC) curves of random forest model for E2 and PPT exposure prediction.

As indicated in Fig. 4A, the raw data collected from Harmony were pre-processed, and OPLS-DA was applied to perform the differential image features analysis for E2 and PPT, respectively. In a cross-comparison, 140 common differential image features were found between the E2 and PPT group, suggesting a similar effect of E2 and PPT on the MCF7 microtissues (Supplemental_Data_11). The 182 E2-associated features, 155 PPT-associated features, and the 140 common features were then used as three separate sets of observations for the following machine learning classification models. The random forests (RF) were utilized as the machine learning algorithm for the classification model construction. The original data were split in an 8:2 ratio randomly, 80% of the data were fed as the training data, and the rest 20% were saved for model validation. The confusion matrixes show that the established model using the 140 common features performed well predicting E2 and PPT at all levels (Fig. 4B). Notably, the prediction results were consistent with the actual grouping for middle and high concentrations of E2 and PPT. The receiver operating characteristic curves (ROC) were a plot to evaluate the performance of different models in estrogen exposure prediction. The upper-left panel of Fig. 4C revealed the performance of the model built with 182 E2-associated features. It performed the best in high-concentration E2 prediction, whereas it did not work well in low-concentration E2 prediction. The AUC for the control, low, medium, and high concentration groups were 0.9361, 0.8831, 0.9080, and 0.9799, respectively, and the AUC_macro-average_ and AUC_micro-average_ were 0.9255 and 0.9377, respectively (Fig. 4C, upper-left panel).

Similarly, PPT prediction with the model using the PPT-associated features predicted high-concentration PPT exposure the best but medium concentration the worst. The AUC for the control, low, medium, and high concentration groups were 0.9045, 0.9189, 0.8885, and 0.9867, respectively, and the AUC_macro-average_ and AUC_mimcro-average_ were 0.9237 and 0.9391, respectively. The model built with the 140 common features was tested for both E2 and PPT. In this model, the AUC_macro-average_ of E2 and PPT were increased to 0.9528 and 0.9513. respectively, which demonstrated an enhanced performance compared to the two models above and suggested that it may serve as a general model of estrogenic effect prediction for a broader spectrum of chemicals (Fig. 4C, lower-left panel).

### MCF7 microtissue lumen characterization by deep learning-assisted 3D lumen analysis for estrogen response prediction

**Figure 5:**
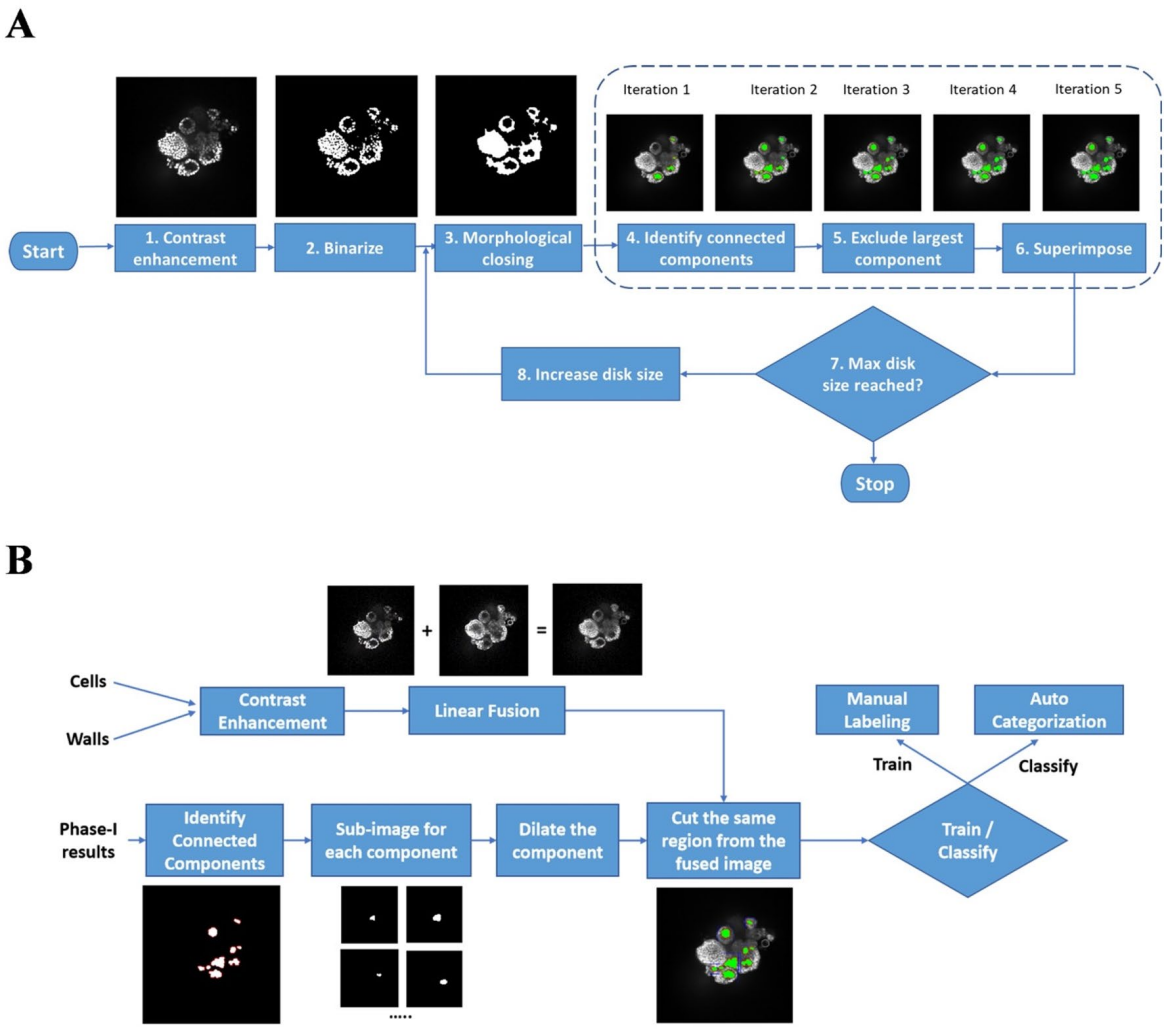
Workflow of constructing the automated lumen identification and analysis software. (**A**) The phase-I image processing pipeline for volumetric analysis of MCF7 lumens; (**B**) The phase-II image processing pipeline for volumetric analysis of MCF7 lumens.

The MCF7 microtissue can automatically form luminal structures, and the number and size of lumens are responsive to estrogen exposure. We built a deep learning-assisted lumen analysis system to characterize the luminal structure in each microtissue. The system has three main components, an image-processing pipeline, a deep learning pipeline and a volumetric analysis step. The system takes stacks of 2D images (z-slices) generated by scanning 3D cell cultures as input, identifies “true” 2D lumens (in the 2D z-slices), re-constructs their corresponding 3D lumens and finally displays and calculates the volumes of the re-constructed 3D lumens. The image processing pipeline initially converts each 2D image (z-slice) to a black and white image. Then, we apply morphological closing (dilation then erosion) to each z-slice to close gaps caused by imperfect imaging. The goal here is to have each potential lumen in the image completely enclosed within its boundaries. At this stage we use a relatively small disk size (6 μm) to avoid the risk of smaller potential lumens being fully closed (thus lost). After closing the gaps, we identify potential lumens by separating out connected components. Since targeted lumens in a z-slice are actually voids, they have the same color as the background. Consequently, we discard the largest connected component (which represents the background). The relatively small disk size that we use in this first iteration will typically miss larger gaps, which means that some lumens with larger gaps in their boundaries will not be detected. Due to these gaps these potential lumens will not be identified as independent connected components, instead they will be captured incorrectly as part of the background. In order to properly capture these potential lumens, we repeat the same procedure (closing, identifying connected components, discarding largest connected component) several times (8 iterations) using gradually increasing disk sizes (5 μm increments). Thus, gradually closing larger and larger gaps. Finally, all connected components from all iterations are superimposed to generate the final set of potential lumens (subject to further classification to determine whether they are “true” lumens or not). Since a potential lumen may have been identified in multiple subsequent iterations with decreasing surface areas (due to the increased disk size), super-imposition not only guarantees that only one copy for each potential lumen is kept, it also makes sure that for each potential lumen, we capture its largest detected form. The outcome of this phase is an image where lumen-like regions are marked (identified), see (Fig. 5A). The images with identified potential lumens are further processed to prepare them for classification (see Fig. 5B). Each identified component is dilated, remapped, and cut from the fused images (the image created by fusing the cells image and the walls image) for true-lumen confirmation. Now, we classify our potential lumens into “true” and “false” lumens (enclosed voids that are not truly lumens). For the purpose of training our classifier, we manually marked about 1000 true lumens to use as a training dataset. We used our training dataset to repurpose a general purpose deep neural network (AlexNet) into a lumen classifier. Finally, we validated our classifier and used it for automatic lumen identification (Fig. 5B).

**Figure 6:**
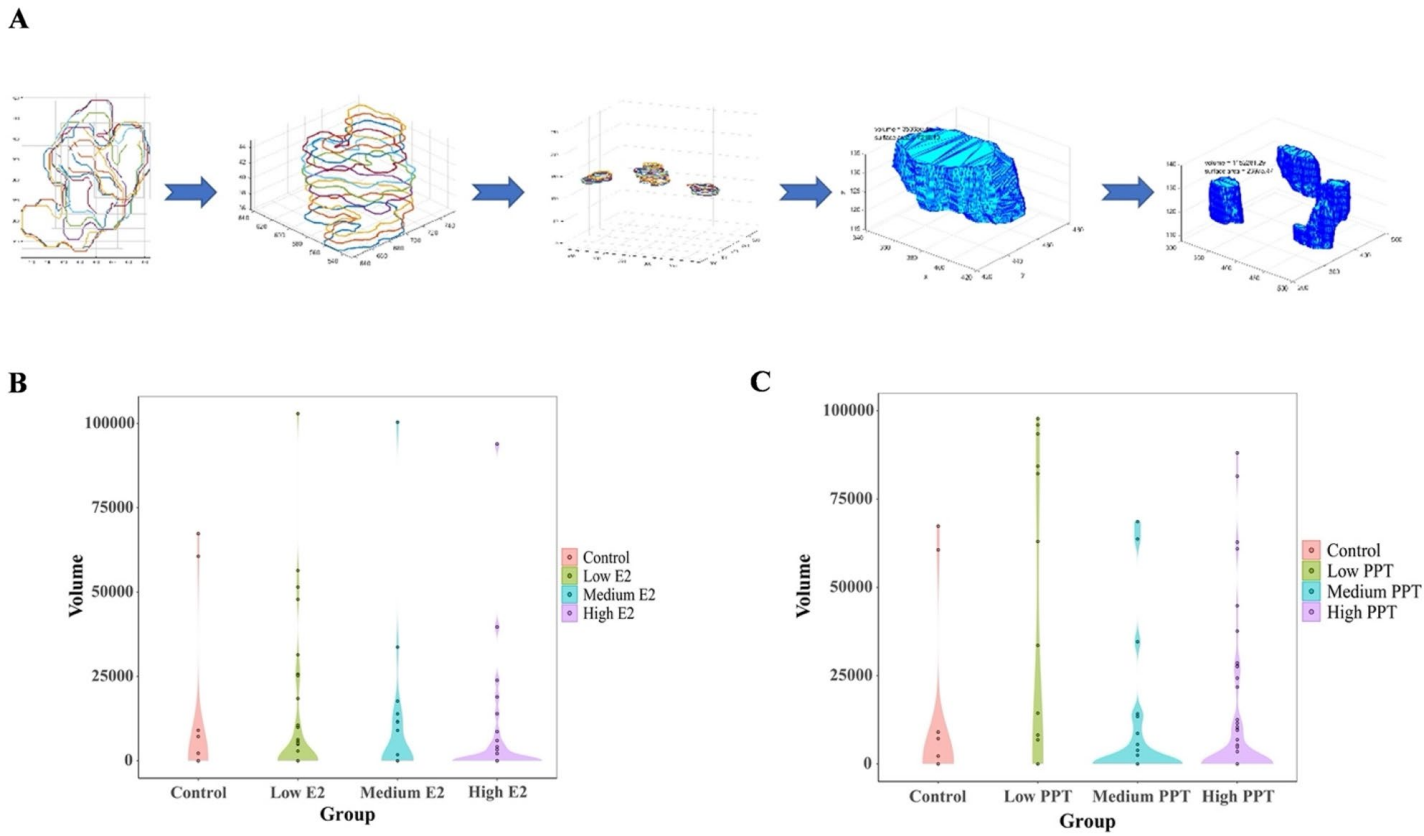
3D luminal space reconstruction and luminal volume analysis. (**A**) Flow chart of volumetric analysis of MCF7 micro tissue lumens; (**B**) Violin plot of MCF7 lumen volume exposed to different concentrations of E2; (**C**) Violin plot of MCF7 lumen volume exposed to varying concentrations of PPT.

Next, we use the “true” (as per our classifier) 2D lumens to reconstruct their corresponding 3D lumens, for further topological analysis. Since a single spheroid (stack of z-slices) may contain more than one lumen (3D lumen), we need to group related 2D lumen, so that each group represents one 3D lumen. Our grouping procedure starts by creating a rectangular bounding box around each identified 2D lumen. Then, we add overlapping 2D lumens (from a vertical view) to the same group (each group of 2D lumens will eventually be used to reconstruct one 3D lumen). It is possible to have gaps within a stack of 2D lumens with overlapping boxes. In such case, we use these gaps to divide the stack into different groups, because in reality it most probably represents several vertically-stacked 3D lumens. Finally, we use the AlphaShape function (MATLAB) to re-construct the 3D lumens, display them and measure their volumes and surface areas (see Fig. 6A). Finally, the system outputs the number of identified lumens and the total luminal volume of each microtissue. It is worth noting that all these steps are fully automated. We applied our 3D lumen volumetric analysis system to the E2 and PPT-exposed microtissues above, and the results were summarized as violin plots in Fig. 6B and C. As shown in Fig. 6B, most of the control microtissues tended to form luminal structures. However, the volume of lumen space was generally small. Compared to the control, more E2-treated microtissues had no lumen detected. However, the ones with detected lumens tended to have larger lumen volumes. These changes were not concentration-dependent. The low E2 group had fewer no-lumen microtissues than the high E2 group and more microtissues with larger luminal space (Fig. 6B). In the PPT experiment, almost all low-PPT microtissues formed lumens, most of which were large volumes. However, in the medium and high PPT groups, the number of microtissue with no lumens increased, and for the detected lumens, the volume tended to decrease (Fig. 6C).

## Discussion

A growing body of evidence supports that EDC exposure contributes to various adverse health outcomes in adults and children. However, the current EDC assessment system is inefficient and time consuming, underscoring the need to develop new assessment tools for EDCs. The current study introduced a novel strategy for estrogenic effect evaluation within an in vitro system incorporating the technology of high-content imaging, morphological profiling, and machine learning.

Physiologically, estrogens are a group of female hormones indispensable for sexual and reproductive function development and maintenance, glucose homeostasis, immune robustness, bone health, cardiovascular health, fertility, and neural systems^27^. Estrogens bind to nuclear and membrane estrogen receptors (ERs), initiating subsequent genomic and non-genomic signaling^28–30^. Estrogenic chemicals directly activate or inhibit estrogenic signaling or indirectly modulate the estrogenic action^31^. Therefore, the assessment of estrogenic chemicals mainly relies on capturing disturbances in the estrogenic signaling and related phenotypical changes in biological systems.

Our previous 3D MCF7 microtissue model has successfully established the association between estrogen exposure and microtissue growth pattern and cellular morphology^14,16^. In the present study, we performed cell counting of 3D microtissues by high-content imaging and an automatic cell counting pipeline. We acquired an EC_50_ for the two model estrogens, E_2_ and PPT, consistent with the previous reports^32–34^. Although the simple endpoint of cell number correlated with estrogen exposure, this monotonic response pattern does not reflect the full complexity of estrogenic responses. To capture this complexity, we used a machine learning assisted high content image analysis approach.

Estrogen is an important mediator of mammary gland morphogenesis. Since our 3D MCF7 model can partially recapitulate human mammary morphology, we aimed to capture a comprehensive set of morphological features of these microtissues and correlate them with estrogenic exposure. We first processed the representative 2D slice images for feature acquisition, which could significantly reduce the computing power and processing time burden. The image feature acquisition pipeline enabled the identification and quantification of approximately 450 distinct full image region and nucleus region features, which generated a numeric morphological fingerprint of each microtissue. Similar to the well-accepted analysis of transcriptomic data, the multi-dimensional data of image profiling was used for sample clustering (Fig. 3), to extract the most significant differential image features in response to E2 and PPT. The leading differential features included categories such as “*image_region_area”* and *“image_region_width”*, which correlated with the microtissue's size and cell count. This suggested that estrogen-driven cell growth patterns have a dominant role in shaping the data from the 2D image analysis, which may mask some of the numerically less significant but biologically important features. This bias might attribute to the method we applied in normalizing the data. Herein, we used the mathematical method to select the most appropriate normalization method to limit the normalization-associate bias. Except for a few dominant features, we also captured many texture features, such as *“image_region_Profile_5.5_SP”* and *“image_region_Profile_4.5_SER.Spot”*, which may be associated with the homogeneity and arrangement of cells in the microtissue and the alteration of nucleus morphology. Despite no apparent connection between all the features and their biological indications, the current profiling data contain comprehensive information regarding various biological processes.

The differential image features largely overlapped between E2 and PPT, indicating a similar response pattern between the two chemicals. We use the differential features from E2, PPT, and their combination to build machine-learning classification models for estrogen concentration prediction. Encouragingly, all three models performed well in the specificity and sensitivity of prediction. The model using the combination of E2 plus PPT overlapping features performed best, suggesting a promising potential for applying it in generalized estrogenic EDC assessment.

The complexity of estrogen signaling has been extensively described and is a classic example of a non-monotonic response due to a feedback loop mechanism. The machine learning-assisted high content analysis approach described above may serve as a practical tool for rapid EDC screening but fail to capture the full complexity of the non-monotonic estrogenic dose response. Therefore, we undertook further analysis of biologically relevant apical endpoints related to mammary gland lumen formation. Since no reliable luminal space analysis tools were readily available, we have developed our own luminal analysis software to automate the process of lumen identification, 3D luminal structure reconstruction, and luminal volume calculation.

Gland formation is a common process in many organs with secretory functions; therefore, besides being used here for mammary lumen analysis, this software can be applied in other biological contexts with value beyond the scope of this study.

The overall estrogen signaling depends on the balance between two nuclear estrogen receptor subtypes, ERα and ERβ^27^. The two receptors share highly homogeneous DNA- and ligand-binding domains but may have different transcriptional activities^35–37^. ERα promotes the estrogen-driven development of the mammary ductal epithelium during puberty^38,39^. The ERα-/- mammary glands show no development beyond a rudimentary ductal system^40^. In breast cancers, ERα activation promotes tumor cell proliferation^41–43^. ERβ contributes to mature mammary glands' homeostasis and growth control^44^. It also exerts tumor-suppressive effects by inhibiting cell proliferation, migration, and invasion and promoting apoptosis^45,46^. Collectively, the mammary gland morphology should be regulated by these ER receptors collaboratively. Chemicals with different receptor selectivity and affinity may associate with different mammary phenotypes. In our current study, compared to E2 (non-selective ER agonist), the cell count dose–response curve for PPT is smooth with a broader liner range (Fig. 1). In terms of the luminal volume, almost all low PPT microtissues form lumens with relatively large volumes, the medium PPT group had the least number of microtissues with lumens and with smaller volumes, whereas the number of microtissues with lumens increased with increased volumes in the high PPT group (Fig. 6). Since PPT is a selective agonist of ERα, then this data indicates that ERα regulates cell growth with a monotonic dose response, but regulates cell differentiation with a non-monotonic dose response. Further, these data support an approach that analyzes multiple phenotypic endpoints, including cell growth and complicated differentiation-related processes like gland formation, to provide an integrated assessment of estrogenic responses.

## Conclusion

The current study provides a novel strategy for estrogenic EDC evaluation based on a 3D MCF7 cell culture system. Applying an image profiling technique to representative cross-sectional images of MCF7 microtissues, we acquired a multi-dimensional matrix with extensive phenotypical information and generated a numeric fingerprint of the morphology of each microtissue, extracting the differential image features in response to estrogen exposure. We trained the machine-learning classification models, which performed well in distinguishing the strength of estrogen response and showed the application potential in generalized estrogenic EDC assessment. We further developed software to characterize microtissue luminal volume. This novel tool provided valuable data for the estrogenic effect in this study and can facilitate the analysis of other biological systems with ductal structures. Our approach captured the complexity of functional ER signaling by combining non-supervised image feature profiling and supervised luminal volume characterization. Further validation of this method with other known estrogenic chemicals will be needed to tune these models and optimize these methods for the EDC risk assessment sequence.

### Supplementary Information

Supplementary Information 1.

Supplementary Information 2.

Supplementary Information 3.

Supplementary Information 4.

Supplementary Information 5.

Supplementary Information 6.

Supplementary Information 7.

Supplementary Information 8.

Supplementary Information 9.

Supplementary Information 10.

Supplementary Information 11.

## Data Availability

Most of the datasets used and/or analysed during the current study are included in this published article and its supplementary files, other data would be available from the corresponding author on reasonable request.
