# Supplementary material for: Machine learning-assisted high-content imaging analysis of 3D MCF7 microtissues for estrogenic effect prediction
Source: Sci Rep. 2024 Feb 6;14:2999. doi: 10.1038/s41598-024-53323-6 (PMC10844358; doi:10.1038/s41598-024-53323-6)
Supplement: Supplementary file 1 — Supplementary Information 1. [file 41598_2024_53323_MOESM1_ESM.pdf]

3D nuclear count

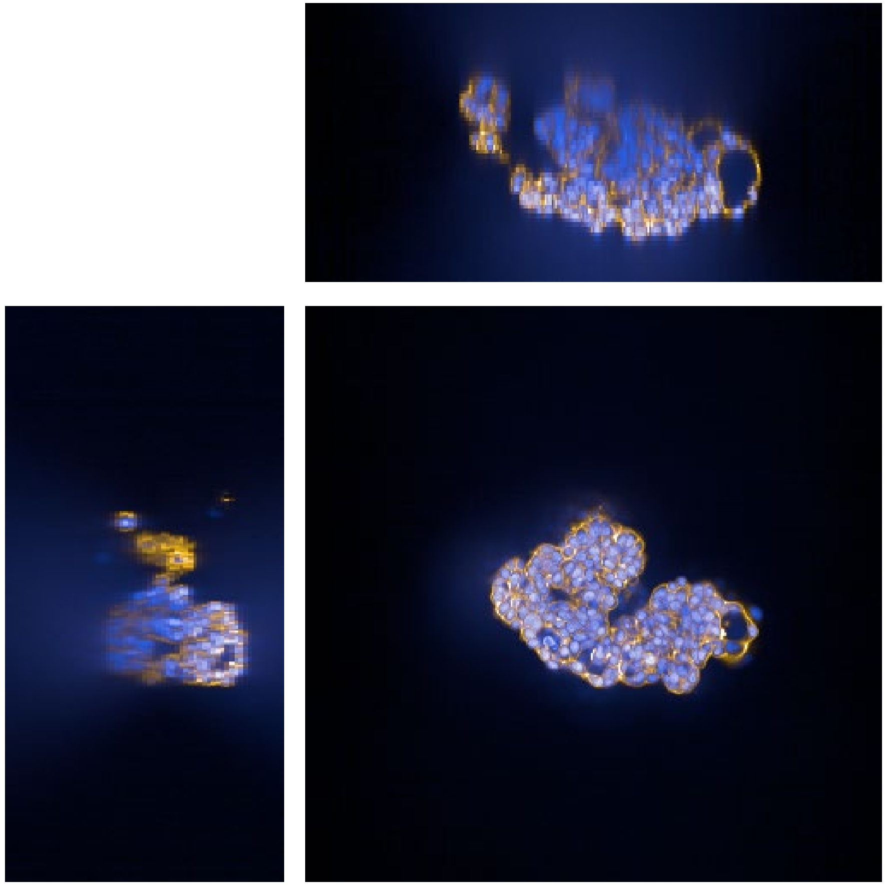

XYZ view

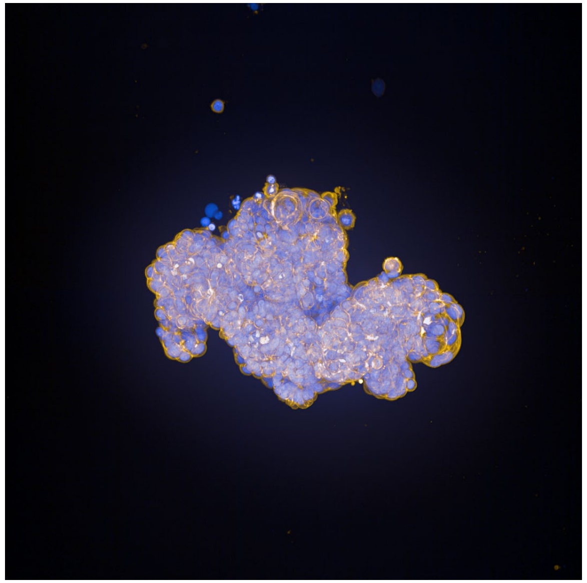

Max projection

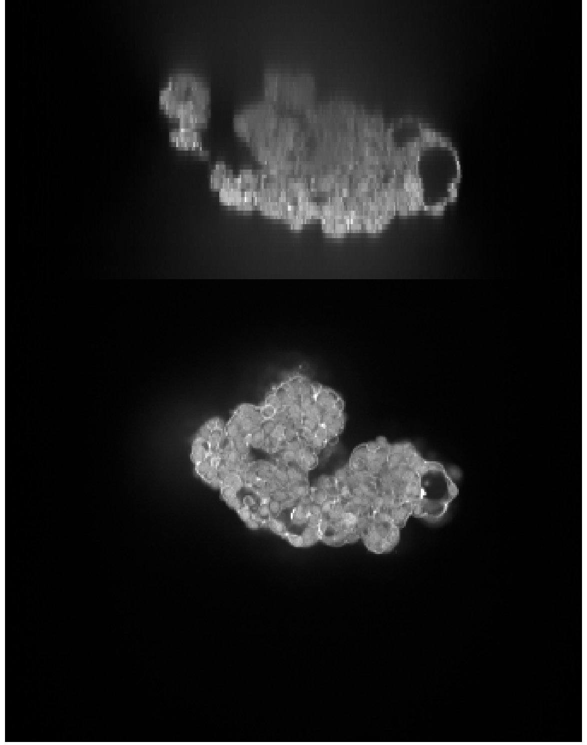

top

1. Calculate Image: Hoechst + rhodamine phalloidin

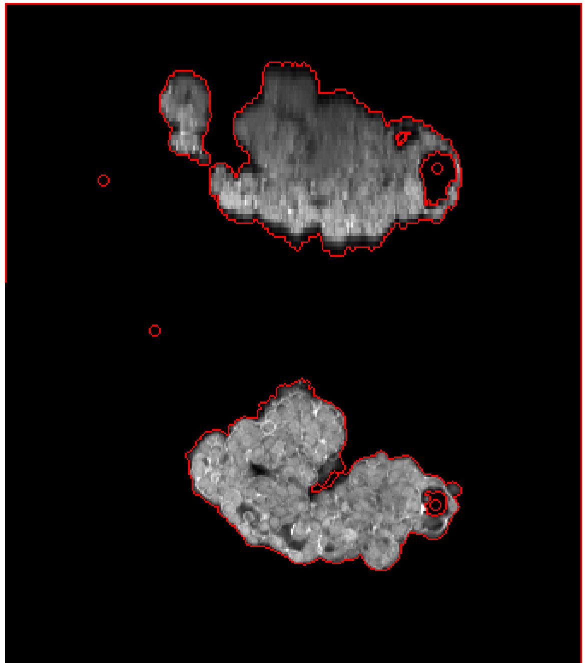

2. Calculate Image: minus background

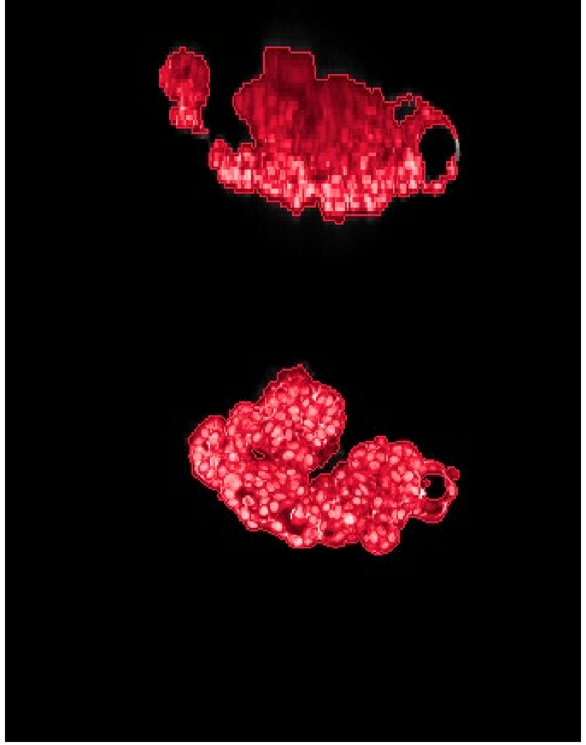

3. Find Image Region: absolute threshold

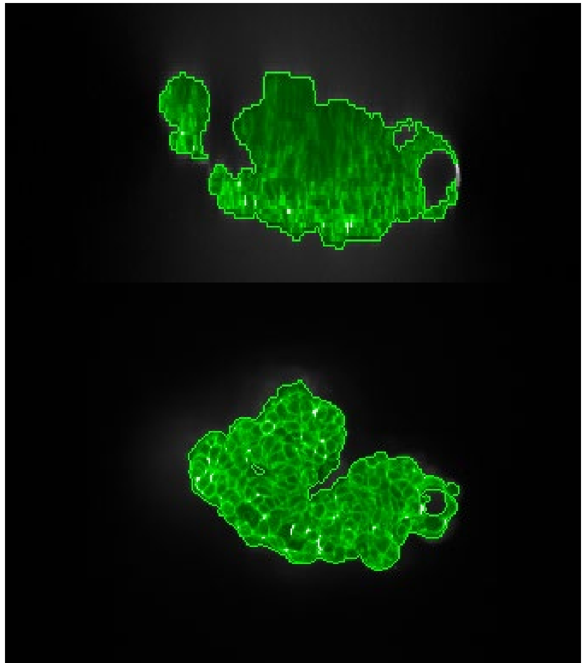

4. Select population: touching side faces

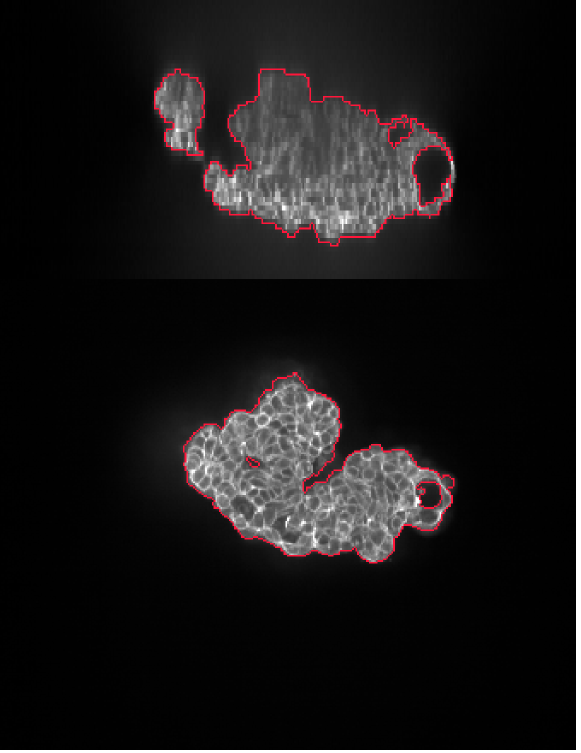

5. Calculate Position Properties:
Nearest neighbor distance

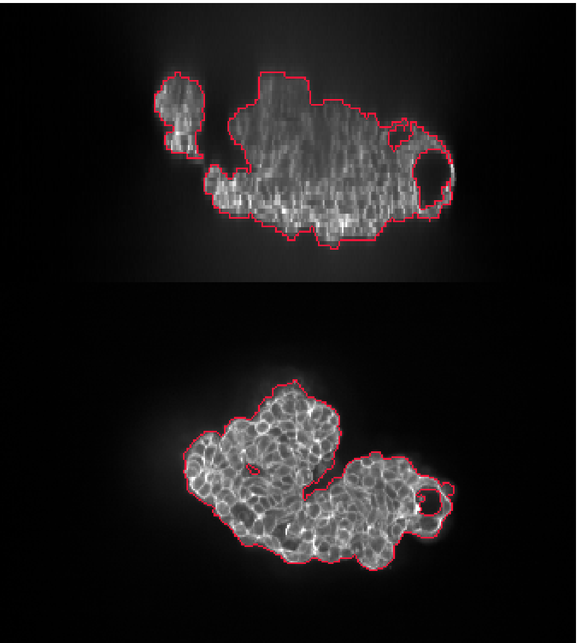

6. Calculate position properties:
Contact area with image border

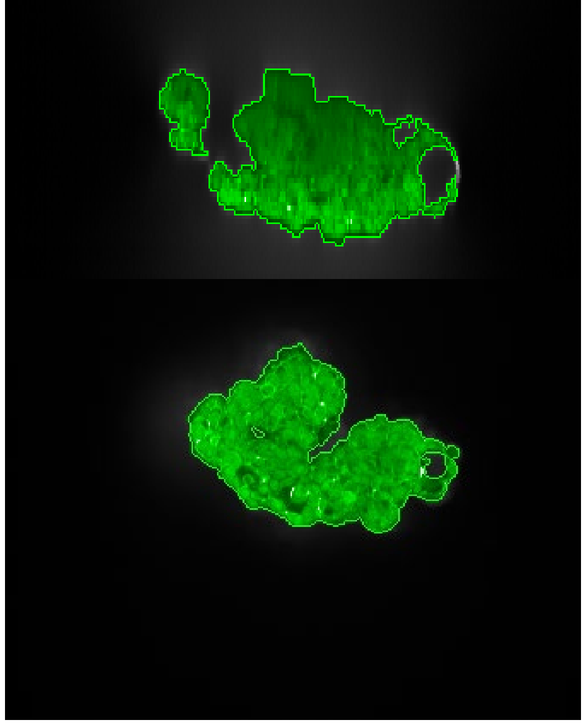

7. Select Population:
filter by property (contact area with bottom and top)

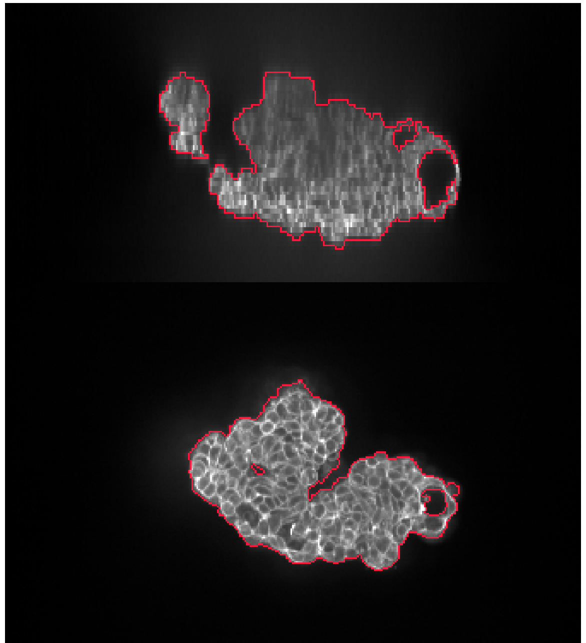

8. Calculate morphology properties
volume

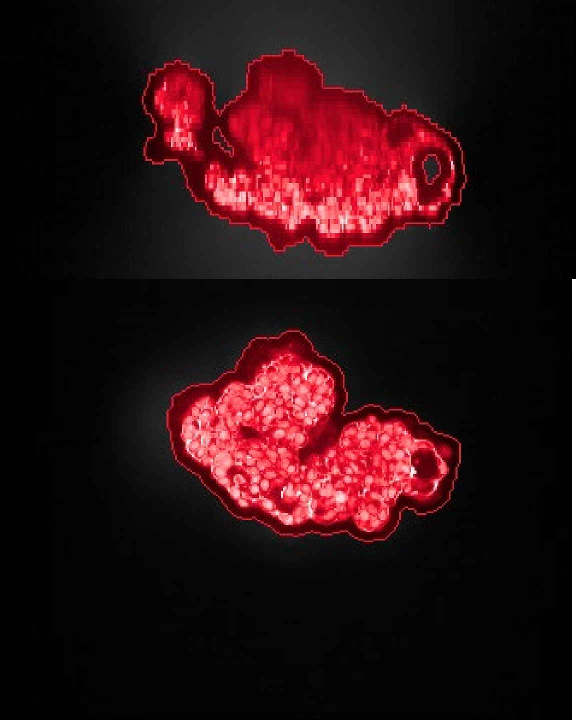

9 Select Region: resize region by 10 uM

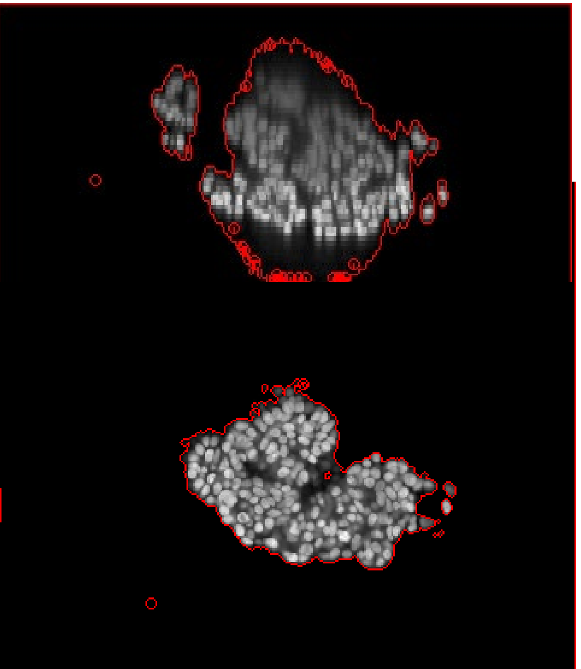

10 Calculate image: remove background from Hoechst

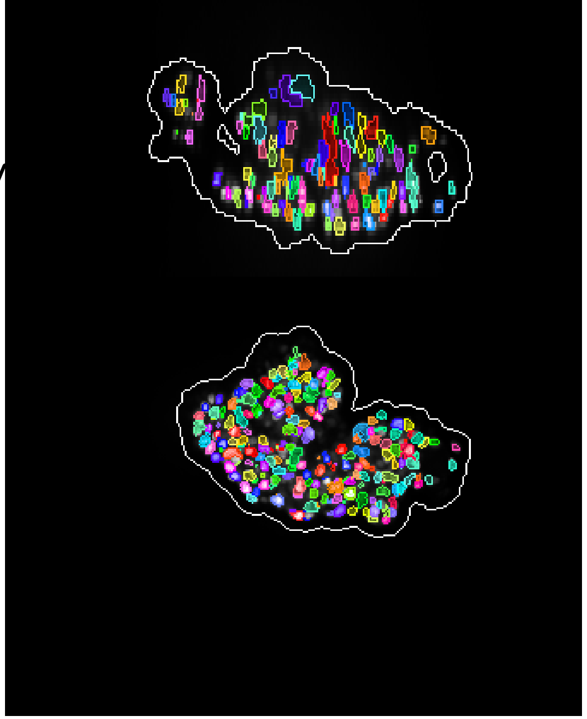

11 find nuclei: method C

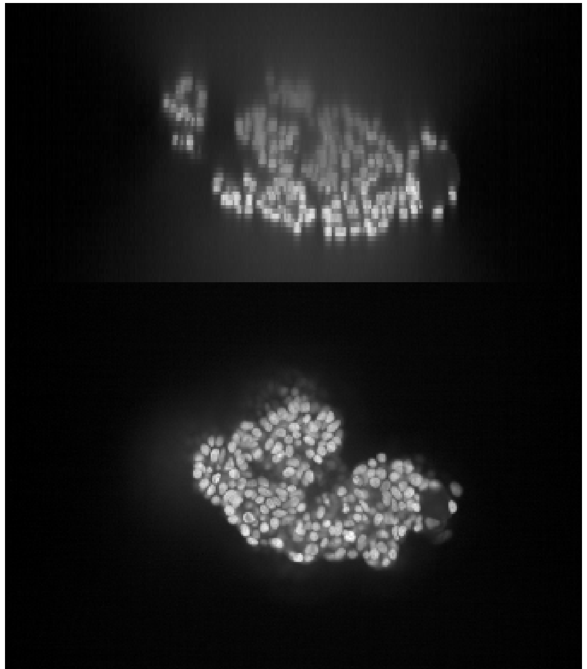

12 calculate morphology properties: volume, object height

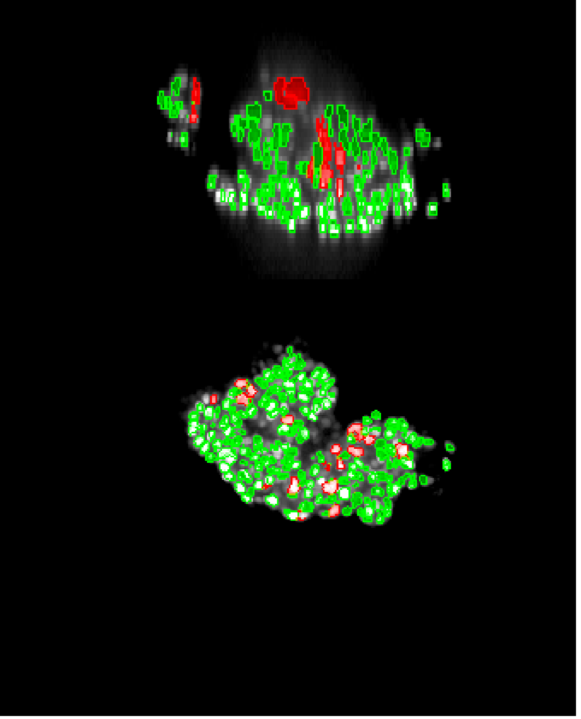

13 Select Population: filter by property (object height, max cross section area, volume)
