# Supplementary material for: Machine learning-assisted high-content imaging analysis of 3D MCF7 microtissues for estrogenic effect prediction
Source: Sci Rep. 2024 Feb 6;14:2999. doi: 10.1038/s41598-024-53323-6 (PMC10844358; doi:10.1038/s41598-024-53323-6)
Supplement: Supplementary file 2 — Supplementary Information 2. [file 41598_2024_53323_MOESM2_ESM.pdf]

Analysis Sequence "n.a."

Input Image	Input
	**Flatfield Correction :** None<br>Brightfield Correction<br>**Stack Processing :** Individual Planes<br>**Min. Global Binning :** Dynamic

Calculate Image	Input	Method	Output
		**Method :** By Formula<br>Formula : A+B<br>Channel A : Alexa 568<br>Channel B : HOECHST 33342<br>Negative Values : Set to Zero<br>Undefined Values : Set to Local Average	Output Image : Calculated Image

Calculate Image (2)	Input	Method	Output
		**Method :** By Formula<br>Formula : A-2000<br>Channel A : Calculated Image<br>Negative Values : Set to Zero<br>Undefined Values : Set to Local Average	Output Image : Calculated Image (2)

Find Image Region	Input	Method	Output
	**Channel :** Calculated Image (2)<br>**ROI :** None	**Method :** Absolute Threshold<br>Lowest Intensity : ≥ 1500<br>Highest Intensity : ≤ inf<br>Split into Objects<br>Area : > 2000 μm²<br>Fill Holes	Output Population : Image Region<br>Output Region : Image Region

Calculate Position Properties	Input	Method	Output
	**Population :** Image Region<br>**Region :** Image Region	**Method :** Standard<br>Nearest Neighbor Distance<br>Contact Area with Neighbors	Property Prefix : Image Region

Select Population (2)	Input	Method	Output
	**Population :** Image Region	**Method :** Common Filters<br>Remove Border Objects<br>Region : Image Region	Output Population : spheroid

Find Image Region (2)	Input	Method	Output
	**Channel :** Calculated Image (2)<br>**ROI :** spheroid<br>**ROI Region :** Image Region	**Method :** Common Threshold<br>Threshold : 0<br>Area : > 0 px²<br>Fill Holes	Output Population : Final spheroid<br>Output Region : Final spheroid

Select Region	Input	Method	Output
	**Population :** Final spheroid<br>**Region :** Final spheroid	**Method :** Resize Region [µm/px]<br>Outer Border : -10 µm<br>Restrictive Population : None<br>Restrictive Region :<br>Inner Border : INF µm	Output Region : Nuclear Region resized

Find Nuclei	Input	Method	Output
	**Channel :** HOECHST 33342<br>**ROI :** Final spheroid<br>**ROI Region :** Nuclear Region resized	**Method :** A<br>Common Threshold : 0.4<br>Area : > 30 µm²<br>Splitting Coefficient : 5<br>Individual Threshold : 0.4<br>Contrast : > 0.1	Output Population : Nuclei

Calculate Morphology Properties	Input	Method	Output
	**Population :** Nuclei<br>**Region :** Nucleus	**Method :** Standard<br>Area<br>Width<br>Length	Property Prefix : Nucleus

Select Population	Input	Method	Output
	**Population :** Nuclei	**Method :** Filter by Property<br>Nucleus Area [µm²] : < 300<br>Nucleus Length [µm] : < 20<br>Boolean Operations : F1 and F2	Output Population : Final Nuclei

Calculate Intensity	Input	Method	Output

Properties			
	**Channel :** Alexa 568<br>**Population :** Final spheroid<br>**Region :** Final spheroid	**Method :** Standard<br>Mean	Property Prefix :<br>Intensity Final spheroid Alexa 568

Calculate Intensity Properties (2)	Input	Method	Output
	**Channel :** HOECHST 33342<br>**Population :** Final spheroid<br>**Region :** Final spheroid	**Method :** Standard<br>Mean	Property Prefix :<br>Intensity Image Region Hoeschst

Calculate Intensity Properties (3)	Input	Method	Output
	**Channel :** HOECHST 33342<br>**Population :** Final Nuclei<br>**Region :** Nucleus	**Method :** Standard<br>Mean	Property Prefix :<br>Intensity Nuclei hoechst

Calculate Morphology Properties (2)	Input	Method	Output
	**Population :** Final spheroid<br>**Region :** Final spheroid	**Method :** Standard<br>Area<br>Roundness<br>Width<br>Length<br>Ratio Width to Length	Property Prefix : Final spheroid

Calculate Morphology Properties (3)	Input	Method	Output
	**Population :** Final spheroid<br>**Region :** Final spheroid	**Method :** STAR<br>Channel : Alexa 568<br>Symmetry<br>Threshold Compactness<br>Axial<br>Radial<br>Profile<br>Profile Inner Region : Nucleus<br>Profile Width : 4 px<br>**Sliding Parabola**<br>Sliding Parabola Curvature : 10<br><br>**Texture SER**<br>Scale : 1 px<br>Normalization by : Kernel<br>SER Spot	Property Prefix : Final spheroid

		SER Hole<br>SER Edge<br>SER Ridge<br>SER Valley<br>SER Saddle<br>SER Bright<br>SER Dark	

**Calculate Texture Properties**	**Input**	**Method**	**Output**
	**Channel :** Alexa 568<br>**Population :** Final spheroid<br>**Region :** Final spheroid	**Method :** SER Features<br>Scale : 1 px<br>Normalization by : Kernel<br>SER Spot<br>SER Hole<br>SER Edge<br>SER Ridge<br>SER Valley<br>SER Saddle<br>SER Bright<br>SER Dark	Property Prefix : Image Region

**Calculate Texture Properties (2)**	**Input**	**Method**	**Output**
	**Channel :** Alexa 568<br>**Population :** Final spheroid<br>**Region :** Final spheroid	**Method :** Haralick Features<br>Distance : 1 px<br>Haralick Contrast<br>Haralick Correlation<br>Haralick Sum Variance<br>Haralick Homogeneity	Property Prefix : Image Region

**Calculate Texture Properties (3)**	**Input**	**Method**	**Output**
	**Channel :** Alexa 568<br>**Population :** Final spheroid<br>**Region :** Final spheroid	**Method :** Gabor Features<br>Scale : 2 px<br>Wavelength : 2<br>Number of Angles : 8<br>Normalization by : Kernel<br>Gabor Min<br>Gabor Max	Property Prefix : Image Region

**Calculate Morphology Properties (4)**	**Input**	**Method**	**Output**
	**Population :** Final Nuclei<br>**Region :** Nucleus	**Method :** Standard<br>Area<br>Roundness<br>Width<br>Length<br>Ratio Width to Length	Property Prefix : Nucleus

Calculate Morphology Properties (5)	Input	Method	Output
	**Population :** Final Nuclei<br>**Region :** Nucleus	**Method :** STAR<br>Channel : HOECHST 33342<br>Symmetry<br>Threshold Compactness<br>Axial<br>Radial<br>Profile<br>Profile Width : 4 px<br>**Sliding Parabola**<br>Sliding Parabola Curvature : 10<br><br>**Texture SER**<br>Scale : 1 px<br>Normalization by : Kernel<br>SER Spot<br>SER Hole<br>SER Edge<br>SER Ridge<br>SER Valley<br>SER Saddle<br>SER Bright<br>SER Dark	Property Prefix : Nucleus

Calculate Texture Properties (5)	Input	Method	Output
	**Channel :** HOECHST 33342<br>**Population :** Final Nuclei<br>**Region :** Nucleus	**Method :** Haralick Features<br>Distance : 1 px<br>Haralick Contrast<br>Haralick Correlation<br>Haralick Sum Variance<br>Haralick Homogeneity	Property Prefix : Nucleus

Calculate Texture Properties (6)	Input	Method	Output
	**Channel :** HOECHST 33342<br>**Population :** Final Nuclei<br>**Region :** Nucleus	**Method :** Gabor Features<br>Scale : 2 px<br>Wavelength : 2<br>Number of Angles : 8<br>Normalization by : Kernel<br>Gabor Min<br>Gabor Max	Property Prefix : Nucleus HOECHST 33342

Calculate Texture Properties (4)	Input	Method	Output
	**Channel :** HOECHST 33342	**Method :** SER Features<br>Scale : 2 px	Property Prefix : Nucleus

	**Population :** Final Nuclei<br>**Region :** Nucleus	Normalization by : Kernel<br>SER Spot<br>SER Hole<br>SER Edge<br>SER Ridge<br>SER Valley<br>SER Saddle<br>SER Bright<br>SER Dark	

**Calculate Properties**	**Input**	**Method**	**Output**
	**Population :** Final spheroid	**Method :** By Related Population<br>Related Population : Final Nuclei<br>Number of Final Nuclei<br>Nucleus Area [μm²]<br>Nucleus Width [μm]<br>Nucleus Length [μm]<br>Intensity Nuclei hoechst Mean<br>Nucleus Area [μm²] (2)<br>Nucleus Roundness<br>Nucleus Width [μm] (2)<br>Nucleus Length [μm] (2)<br>Nucleus Ratio Width to Length<br>Nucleus Symmetry 02<br>Nucleus Symmetry 03<br>Nucleus Symmetry 04<br>Nucleus Symmetry 05<br>Nucleus Symmetry 12<br>Nucleus Symmetry 13<br>Nucleus Symmetry 14<br>Nucleus Symmetry 15<br>Nucleus Threshold Compactness 30%<br>Nucleus Threshold Compactness 40%<br>Nucleus Threshold Compactness 50%<br>Nucleus Threshold Compactness 60%<br>Nucleus Axial Small Length<br>Nucleus Axial Length Ratio<br>Nucleus Radial Mean<br>Nucleus Radial Relative Deviation<br>Nucleus Profile 1/2<br>Nucleus Profile 2/2<br>Nucleus Symmetry 02 SP-Filter<br>Nucleus Symmetry 03 SP-Filter<br>Nucleus Symmetry 04 SP-Filter<br>Nucleus Symmetry 05 SP-Filter	Property Suffix : per Object

		Nucleus Symmetry 12 SP-Filter<br>Nucleus Symmetry 13 SP-Filter<br>Nucleus Symmetry 14 SP-Filter<br>Nucleus Symmetry 15 SP-Filter<br>Nucleus Threshold Compactness 30% SP-Filter<br>Nucleus Threshold Compactness 40% SP-Filter<br>Nucleus Threshold Compactness 50% SP-Filter<br>Nucleus Threshold Compactness 60% SP-Filter<br>Nucleus Axial Small Length SP-Filter<br>Nucleus Axial Length Ratio SP-Filter<br>Nucleus Radial Mean SP-Filter<br>Nucleus Radial Relative Deviation SP-Filter<br>Nucleus Radial Mean Ratio SP-Filter<br>Nucleus Profile 1/2 SP-Filter<br>Nucleus Profile 2/2 SP-Filter<br>Nucleus Symmetry 02 SER-Bright<br>Nucleus Symmetry 03 SER-Bright<br>Nucleus Symmetry 04 SER-Bright<br>Nucleus Symmetry 05 SER-Bright<br>Nucleus Symmetry 12 SER-Bright<br>Nucleus Symmetry 13 SER-Bright<br>Nucleus Symmetry 14 SER-Bright<br>Nucleus Symmetry 15 SER-Bright<br>Nucleus Threshold Compactness 30% SER-Bright<br>Nucleus Threshold Compactness 40% SER-Bright<br>Nucleus Threshold Compactness 50% SER-Bright<br>Nucleus Threshold Compactness 60% SER-	

		Bright<br>Nucleus Axial Small Length SER-Bright<br>Nucleus Axial Length Ratio SER-Bright<br>Nucleus Radial Mean SER-Bright<br>Nucleus Radial Relative Deviation SER-Bright<br>Nucleus Radial Mean Ratio SER-Bright<br>Nucleus Profile 1/2 SER-Bright<br>Nucleus Profile 2/2 SER-Bright<br>Nucleus Symmetry 02 SER-Dark<br>Nucleus Symmetry 03 SER-Dark<br>Nucleus Symmetry 04 SER-Dark<br>Nucleus Symmetry 05 SER-Dark<br>Nucleus Symmetry 12 SER-Dark<br>Nucleus Symmetry 13 SER-Dark<br>Nucleus Symmetry 14 SER-Dark<br>Nucleus Symmetry 15 SER-Dark<br>Nucleus Threshold Compactness 30% SER-Dark<br>Nucleus Threshold Compactness 40% SER-Dark<br>Nucleus Threshold Compactness 50% SER-Dark<br>Nucleus Threshold Compactness 60% SER-Dark<br>Nucleus Axial Small Length SER-Dark<br>Nucleus Axial Length Ratio SER-Dark<br>Nucleus Radial Mean SER-Dark<br>Nucleus Radial Relative Deviation SER-Dark<br>Nucleus Radial Mean Ratio SER-Dark<br>Nucleus Profile 1/2 SER-Dark<br>Nucleus Profile 2/2 SER-Dark<br>Nucleus Symmetry 02 SER-Ridge<br>Nucleus Symmetry 03 SER-Ridge	

		Nucleus Symmetry 04 SER-Ridge<br>Nucleus Symmetry 05 SER-Ridge<br>Nucleus Symmetry 12 SER-Ridge<br>Nucleus Symmetry 13 SER-Ridge<br>Nucleus Symmetry 14 SER-Ridge<br>Nucleus Symmetry 15 SER-Ridge<br>Nucleus Threshold Compactness 30% SER-Ridge<br>Nucleus Threshold Compactness 40% SER-Ridge<br>Nucleus Threshold Compactness 50% SER-Ridge<br>Nucleus Threshold Compactness 60% SER-Ridge<br>Nucleus Axial Small Length SER-Ridge<br>Nucleus Axial Length Ratio SER-Ridge<br>Nucleus Radial Mean SER-Ridge<br>Nucleus Radial Relative Deviation SER-Ridge<br>Nucleus Radial Mean Ratio SER-Ridge<br>Nucleus Profile 1/2 SER-Ridge<br>Nucleus Profile 2/2 SER-Ridge<br>Nucleus Symmetry 02 SER-Valley<br>Nucleus Symmetry 03 SER-Valley<br>Nucleus Symmetry 04 SER-Valley<br>Nucleus Symmetry 05 SER-Valley<br>Nucleus Symmetry 12 SER-Valley<br>Nucleus Symmetry 13 SER-Valley<br>Nucleus Symmetry 14 SER-Valley<br>Nucleus Symmetry 15 SER-Valley<br>Nucleus Threshold Compactness 30% SER-Valley<br>Nucleus Threshold Compactness 40% SER-Valley	

		Nucleus Threshold Compactness 50% SER-Valley<br>Nucleus Threshold Compactness 60% SER-Valley<br>Nucleus Axial Small Length SER-Valley<br>Nucleus Axial Length Ratio SER-Valley<br>Nucleus Radial Mean SER-Valley<br>Nucleus Radial Relative Deviation SER-Valley<br>Nucleus Radial Mean Ratio SER-Valley<br>Nucleus Profile 1/2 SER-Valley<br>Nucleus Profile 2/2 SER-Valley<br>Nucleus Symmetry 02 SER-Spot<br>Nucleus Symmetry 03 SER-Spot<br>Nucleus Symmetry 04 SER-Spot<br>Nucleus Symmetry 05 SER-Spot<br>Nucleus Symmetry 12 SER-Spot<br>Nucleus Symmetry 13 SER-Spot<br>Nucleus Symmetry 14 SER-Spot<br>Nucleus Symmetry 15 SER-Spot<br>Nucleus Threshold Compactness 30% SER-Spot<br>Nucleus Threshold Compactness 40% SER-Spot<br>Nucleus Threshold Compactness 50% SER-Spot<br>Nucleus Threshold Compactness 60% SER-Spot<br>Nucleus Axial Small Length SER-Spot<br>Nucleus Axial Length Ratio SER-Spot<br>Nucleus Radial Mean SER-Spot<br>Nucleus Radial Relative Deviation SER-Spot<br>Nucleus Radial Mean Ratio SER-Spot<br>Nucleus Profile 1/2 SER-Spot	

		Nucleus Profile 2/2 SER-Spot<br>Nucleus Symmetry 02 SER-Hole<br>Nucleus Symmetry 03 SER-Hole<br>Nucleus Symmetry 04 SER-Hole<br>Nucleus Symmetry 05 SER-Hole<br>Nucleus Symmetry 12 SER-Hole<br>Nucleus Symmetry 13 SER-Hole<br>Nucleus Symmetry 14 SER-Hole<br>Nucleus Symmetry 15 SER-Hole<br>Nucleus Threshold Compactness 30% SER-Hole<br>Nucleus Threshold Compactness 40% SER-Hole<br>Nucleus Threshold Compactness 50% SER-Hole<br>Nucleus Threshold Compactness 60% SER-Hole<br>Nucleus Axial Small Length SER-Hole<br>Nucleus Axial Length Ratio SER-Hole<br>Nucleus Radial Mean SER-Hole<br>Nucleus Radial Relative Deviation SER-Hole<br>Nucleus Radial Mean Ratio SER-Hole<br>Nucleus Profile 1/2 SER-Hole<br>Nucleus Profile 2/2 SER-Hole<br>Nucleus Symmetry 02 SER-Saddle<br>Nucleus Symmetry 03 SER-Saddle<br>Nucleus Symmetry 04 SER-Saddle<br>Nucleus Symmetry 05 SER-Saddle<br>Nucleus Symmetry 12 SER-Saddle<br>Nucleus Symmetry 13 SER-Saddle<br>Nucleus Symmetry 14 SER-Saddle<br>Nucleus Symmetry 15 SER-Saddle	

		Nucleus Threshold Compactness 30% SER-Saddle<br>Nucleus Threshold Compactness 40% SER-Saddle<br>Nucleus Threshold Compactness 50% SER-Saddle<br>Nucleus Threshold Compactness 60% SER-Saddle<br>Nucleus Axial Small Length SER-Saddle<br>Nucleus Axial Length Ratio SER-Saddle<br>Nucleus Radial Mean SER-Saddle<br>Nucleus Radial Relative Deviation SER-Saddle<br>Nucleus Radial Mean Ratio SER-Saddle<br>Nucleus Profile 1/2 SER-Saddle<br>Nucleus Profile 2/2 SER-Saddle<br>Nucleus Symmetry 02 SER-Edge<br>Nucleus Symmetry 03 SER-Edge<br>Nucleus Symmetry 04 SER-Edge<br>Nucleus Symmetry 05 SER-Edge<br>Nucleus Symmetry 12 SER-Edge<br>Nucleus Symmetry 13 SER-Edge<br>Nucleus Symmetry 14 SER-Edge<br>Nucleus Symmetry 15 SER-Edge<br>Nucleus Threshold Compactness 30% SER-Edge<br>Nucleus Threshold Compactness 40% SER-Edge<br>Nucleus Threshold Compactness 50% SER-Edge<br>Nucleus Threshold Compactness 60% SER-Edge<br>Nucleus Axial Small Length SER-Edge<br>Nucleus Axial Length Ratio SER-Edge<br>Nucleus Radial Mean SER-Edge	

		Nucleus Radial Relative Deviation SER-Edge<br>Nucleus Radial Mean Ratio SER-Edge<br>Nucleus Profile 1/2 SER-Edge<br>Nucleus Profile 2/2 SER-Edge<br>Nucleus Haralick Correlation 1 px<br>Nucleus Haralick Contrast 1 px<br>Nucleus Haralick Sum Variance 1 px<br>Nucleus Haralick Homogeneity 1 px<br>Nucleus HOECHST 33342 Gabor Min 2 px w2<br>Nucleus HOECHST 33342 Gabor Max 2 px w2<br>Nucleus SER Spot 2 px<br>Nucleus SER Hole 2 px<br>Nucleus SER Edge 2 px<br>Nucleus SER Ridge 2 px<br>Nucleus SER Valley 2 px<br>Nucleus SER Saddle 2 px<br>Nucleus SER Bright 2 px<br>Nucleus SER Dark 2 px	

Define Results	Results
	**Method :** List of Outputs<br>**Population : Image Region**<br>Apply to All : None<br><br>**Population : Final Nuclei**<br>Apply to All : None<br><br>**Population : Final spheroid**<br>Number of Objects<br>Apply to All : Mean<br>Intensity Final spheroid Alexa 568 Mean : Mean<br>Intensity Image Region Hoeschst Mean : Mean<br>Final spheroid Area [µm²] : Mean<br>Final spheroid Roundness : Mean<br>Final spheroid Width [µm] : Mean<br>Final spheroid Length [µm] : Mean<br>Final spheroid Ratio Width to Length : Mean<br>STAR Properties (Final spheroid) : Mean<br>Image Region SER Spot 1 px : Mean<br>Image Region SER Hole 1 px : Mean<br>Image Region SER Edge 1 px : Mean<br>Image Region SER Ridge 1 px : Mean<br>Image Region SER Valley 1 px : Mean<br>Image Region SER Saddle 1 px : Mean<br>Image Region SER Bright 1 px : Mean<br>Image Region SER Dark 1 px : Mean<br>Image Region Haralick Correlation 1 px : Mean<br>Image Region Haralick Contrast 1 px : Mean<br>Image Region Haralick Sum Variance 1 px : Mean

Image Region Haralick Homogeneity 1 px : Mean
Image Region Gabor Min 2 px w2 : Mean
Image Region Gabor Max 2 px w2 : Mean
Number of Final Nuclei- per Object : Mean
Nucleus Area [μm²]- Mean per Object : Mean
Nucleus Width [μm]- Mean per Object : Mean
Nucleus Length [μm]- Mean per Object : Mean
Intensity Nuclei hoechst Mean- Mean per Object : Mean
Nucleus Area [μm²] (2)- Mean per Object : Mean
Nucleus Roundness- Mean per Object : Mean
Nucleus Width [μm] (2)- Mean per Object : Mean
Nucleus Length [μm] (2)- Mean per Object : Mean
Nucleus Ratio Width to Length- Mean per Object : Mean
Nucleus Symmetry 02 - Mean per Object : Mean
Nucleus Symmetry 03 - Mean per Object : Mean
Nucleus Symmetry 04 - Mean per Object : Mean
Nucleus Symmetry 05 - Mean per Object : Mean
Nucleus Symmetry 12 - Mean per Object : Mean
Nucleus Symmetry 13 - Mean per Object : Mean
Nucleus Symmetry 14 - Mean per Object : Mean
Nucleus Symmetry 15 - Mean per Object : Mean
Nucleus Threshold Compactness 30% - Mean per Object : Mean
Nucleus Threshold Compactness 40% - Mean per Object : Mean
Nucleus Threshold Compactness 50% - Mean per Object : Mean
Nucleus Threshold Compactness 60% - Mean per Object : Mean
Nucleus Axial Small Length - Mean per Object : Mean
Nucleus Axial Length Ratio - Mean per Object : Mean
Nucleus Radial Mean - Mean per Object : Mean
Nucleus Radial Relative Deviation - Mean per Object : Mean
Nucleus Profile 1/2 - Mean per Object : Mean
Nucleus Profile 2/2 - Mean per Object : Mean
Nucleus Symmetry 02 SP-Filter- Mean per Object : Mean
Nucleus Symmetry 03 SP-Filter- Mean per Object : Mean
Nucleus Symmetry 04 SP-Filter- Mean per Object : Mean
Nucleus Symmetry 05 SP-Filter- Mean per Object : Mean
Nucleus Symmetry 12 SP-Filter- Mean per Object : Mean
Nucleus Symmetry 13 SP-Filter- Mean per Object : Mean
Nucleus Symmetry 14 SP-Filter- Mean per Object : Mean
Nucleus Symmetry 15 SP-Filter- Mean per Object : Mean
Nucleus Threshold Compactness 30% SP-Filter- Mean per Object : Mean
Nucleus Threshold Compactness 40% SP-Filter- Mean per Object : Mean
Nucleus Threshold Compactness 50% SP-Filter- Mean per Object : Mean
Nucleus Threshold Compactness 60% SP-Filter- Mean per Object : Mean
Nucleus Axial Small Length SP-Filter- Mean per Object : Mean
Nucleus Axial Length Ratio SP-Filter- Mean per Object : Mean
Nucleus Radial Mean SP-Filter- Mean per Object : Mean
Nucleus Radial Relative Deviation SP-Filter- Mean per Object : Mean
Nucleus Radial Mean Ratio SP-Filter- Mean per Object : Mean
Nucleus Profile 1/2 SP-Filter- Mean per Object : Mean
Nucleus Profile 2/2 SP-Filter- Mean per Object : Mean
Nucleus Symmetry 02 SER-Bright- Mean per Object : Mean
Nucleus Symmetry 03 SER-Bright- Mean per Object : Mean
Nucleus Symmetry 04 SER-Bright- Mean per Object : Mean
Nucleus Symmetry 05 SER-Bright- Mean per Object : Mean
Nucleus Symmetry 12 SER-Bright- Mean per Object : Mean
Nucleus Symmetry 13 SER-Bright- Mean per Object : Mean
Nucleus Symmetry 14 SER-Bright- Mean per Object : Mean
Nucleus Symmetry 15 SER-Bright- Mean per Object : Mean
Nucleus Threshold Compactness 30% SER-Bright- Mean per Object : Mean
Nucleus Threshold Compactness 40% SER-Bright- Mean per Object : Mean
Nucleus Threshold Compactness 50% SER-Bright- Mean per Object : Mean

Nucleus Threshold Compactness 60% SER-Bright- Mean per Object : Mean
Nucleus Axial Small Length SER-Bright- Mean per Object : Mean
Nucleus Axial Length Ratio SER-Bright- Mean per Object : Mean
Nucleus Radial Mean SER-Bright- Mean per Object : Mean
Nucleus Radial Relative Deviation SER-Bright- Mean per Object : Mean
Nucleus Radial Mean Ratio SER-Bright- Mean per Object : Mean
Nucleus Profile 1/2 SER-Bright- Mean per Object : Mean
Nucleus Profile 2/2 SER-Bright- Mean per Object : Mean
Nucleus Symmetry 02 SER-Dark- Mean per Object : Mean
Nucleus Symmetry 03 SER-Dark- Mean per Object : Mean
Nucleus Symmetry 04 SER-Dark- Mean per Object : Mean
Nucleus Symmetry 05 SER-Dark- Mean per Object : Mean
Nucleus Symmetry 12 SER-Dark- Mean per Object : Mean
Nucleus Symmetry 13 SER-Dark- Mean per Object : Mean
Nucleus Symmetry 14 SER-Dark- Mean per Object : Mean
Nucleus Symmetry 15 SER-Dark- Mean per Object : Mean
Nucleus Threshold Compactness 30% SER-Dark- Mean per Object : Mean
Nucleus Threshold Compactness 40% SER-Dark- Mean per Object : Mean
Nucleus Threshold Compactness 50% SER-Dark- Mean per Object : Mean
Nucleus Threshold Compactness 60% SER-Dark- Mean per Object : Mean
Nucleus Axial Small Length SER-Dark- Mean per Object : Mean
Nucleus Axial Length Ratio SER-Dark- Mean per Object : Mean
Nucleus Radial Mean SER-Dark- Mean per Object : Mean
Nucleus Radial Relative Deviation SER-Dark- Mean per Object : Mean
Nucleus Radial Mean Ratio SER-Dark- Mean per Object : Mean
Nucleus Profile 1/2 SER-Dark- Mean per Object : Mean
Nucleus Profile 2/2 SER-Dark- Mean per Object : Mean
Nucleus Symmetry 02 SER-Ridge- Mean per Object : Mean
Nucleus Symmetry 03 SER-Ridge- Mean per Object : Mean
Nucleus Symmetry 04 SER-Ridge- Mean per Object : Mean
Nucleus Symmetry 05 SER-Ridge- Mean per Object : Mean
Nucleus Symmetry 12 SER-Ridge- Mean per Object : Mean
Nucleus Symmetry 13 SER-Ridge- Mean per Object : Mean
Nucleus Symmetry 14 SER-Ridge- Mean per Object : Mean
Nucleus Symmetry 15 SER-Ridge- Mean per Object : Mean
Nucleus Threshold Compactness 30% SER-Ridge- Mean per Object : Mean
Nucleus Threshold Compactness 40% SER-Ridge- Mean per Object : Mean
Nucleus Threshold Compactness 50% SER-Ridge- Mean per Object : Mean
Nucleus Threshold Compactness 60% SER-Ridge- Mean per Object : Mean
Nucleus Axial Small Length SER-Ridge- Mean per Object : Mean
Nucleus Axial Length Ratio SER-Ridge- Mean per Object : Mean
Nucleus Radial Mean SER-Ridge- Mean per Object : Mean
Nucleus Radial Relative Deviation SER-Ridge- Mean per Object : Mean
Nucleus Radial Mean Ratio SER-Ridge- Mean per Object : Mean
Nucleus Profile 1/2 SER-Ridge- Mean per Object : Mean
Nucleus Profile 2/2 SER-Ridge- Mean per Object : Mean
Nucleus Symmetry 02 SER-Valley- Mean per Object : Mean
Nucleus Symmetry 03 SER-Valley- Mean per Object : Mean
Nucleus Symmetry 04 SER-Valley- Mean per Object : Mean
Nucleus Symmetry 05 SER-Valley- Mean per Object : Mean
Nucleus Symmetry 12 SER-Valley- Mean per Object : Mean
Nucleus Symmetry 13 SER-Valley- Mean per Object : Mean
Nucleus Symmetry 14 SER-Valley- Mean per Object : Mean
Nucleus Symmetry 15 SER-Valley- Mean per Object : Mean
Nucleus Threshold Compactness 30% SER-Valley- Mean per Object : Mean
Nucleus Threshold Compactness 40% SER-Valley- Mean per Object : Mean
Nucleus Threshold Compactness 50% SER-Valley- Mean per Object : Mean
Nucleus Threshold Compactness 60% SER-Valley- Mean per Object : Mean
Nucleus Axial Small Length SER-Valley- Mean per Object : Mean
Nucleus Axial Length Ratio SER-Valley- Mean per Object : Mean
Nucleus Radial Mean SER-Valley- Mean per Object : Mean

Nucleus Radial Relative Deviation SER-Valley- Mean per Object : Mean
Nucleus Radial Mean Ratio SER-Valley- Mean per Object : Mean
Nucleus Profile 1/2 SER-Valley- Mean per Object : Mean
Nucleus Profile 2/2 SER-Valley- Mean per Object : Mean
Nucleus Symmetry 02 SER-Spot- Mean per Object : Mean
Nucleus Symmetry 03 SER-Spot- Mean per Object : Mean
Nucleus Symmetry 04 SER-Spot- Mean per Object : Mean
Nucleus Symmetry 05 SER-Spot- Mean per Object : Mean
Nucleus Symmetry 12 SER-Spot- Mean per Object : Mean
Nucleus Symmetry 13 SER-Spot- Mean per Object : Mean
Nucleus Symmetry 14 SER-Spot- Mean per Object : Mean
Nucleus Symmetry 15 SER-Spot- Mean per Object : Mean
Nucleus Threshold Compactness 30% SER-Spot- Mean per Object : Mean
Nucleus Threshold Compactness 40% SER-Spot- Mean per Object : Mean
Nucleus Threshold Compactness 50% SER-Spot- Mean per Object : Mean
Nucleus Threshold Compactness 60% SER-Spot- Mean per Object : Mean
Nucleus Axial Small Length SER-Spot- Mean per Object : Mean
Nucleus Axial Length Ratio SER-Spot- Mean per Object : Mean
Nucleus Radial Mean SER-Spot- Mean per Object : Mean
Nucleus Radial Relative Deviation SER-Spot- Mean per Object : Mean
Nucleus Radial Mean Ratio SER-Spot- Mean per Object : Mean
Nucleus Profile 1/2 SER-Spot- Mean per Object : Mean
Nucleus Profile 2/2 SER-Spot- Mean per Object : Mean
Nucleus Symmetry 02 SER-Hole- Mean per Object : Mean
Nucleus Symmetry 03 SER-Hole- Mean per Object : Mean
Nucleus Symmetry 04 SER-Hole- Mean per Object : Mean
Nucleus Symmetry 05 SER-Hole- Mean per Object : Mean
Nucleus Symmetry 12 SER-Hole- Mean per Object : Mean
Nucleus Symmetry 13 SER-Hole- Mean per Object : Mean
Nucleus Symmetry 14 SER-Hole- Mean per Object : Mean
Nucleus Symmetry 15 SER-Hole- Mean per Object : Mean
Nucleus Threshold Compactness 30% SER-Hole- Mean per Object : Mean
Nucleus Threshold Compactness 40% SER-Hole- Mean per Object : Mean
Nucleus Threshold Compactness 50% SER-Hole- Mean per Object : Mean
Nucleus Threshold Compactness 60% SER-Hole- Mean per Object : Mean
Nucleus Axial Small Length SER-Hole- Mean per Object : Mean
Nucleus Axial Length Ratio SER-Hole- Mean per Object : Mean
Nucleus Radial Mean SER-Hole- Mean per Object : Mean
Nucleus Radial Relative Deviation SER-Hole- Mean per Object : Mean
Nucleus Radial Mean Ratio SER-Hole- Mean per Object : Mean
Nucleus Profile 1/2 SER-Hole- Mean per Object : Mean
Nucleus Profile 2/2 SER-Hole- Mean per Object : Mean
Nucleus Symmetry 02 SER-Saddle- Mean per Object : Mean
Nucleus Symmetry 03 SER-Saddle- Mean per Object : Mean
Nucleus Symmetry 04 SER-Saddle- Mean per Object : Mean
Nucleus Symmetry 05 SER-Saddle- Mean per Object : Mean
Nucleus Symmetry 12 SER-Saddle- Mean per Object : Mean
Nucleus Symmetry 13 SER-Saddle- Mean per Object : Mean
Nucleus Symmetry 14 SER-Saddle- Mean per Object : Mean
Nucleus Symmetry 15 SER-Saddle- Mean per Object : Mean
Nucleus Threshold Compactness 30% SER-Saddle- Mean per Object : Mean
Nucleus Threshold Compactness 40% SER-Saddle- Mean per Object : Mean
Nucleus Threshold Compactness 50% SER-Saddle- Mean per Object : Mean
Nucleus Threshold Compactness 60% SER-Saddle- Mean per Object : Mean
Nucleus Axial Small Length SER-Saddle- Mean per Object : Mean
Nucleus Axial Length Ratio SER-Saddle- Mean per Object : Mean
Nucleus Radial Mean SER-Saddle- Mean per Object : Mean
Nucleus Radial Relative Deviation SER-Saddle- Mean per Object : Mean
Nucleus Radial Mean Ratio SER-Saddle- Mean per Object : Mean
Nucleus Profile 1/2 SER-Saddle- Mean per Object : Mean
Nucleus Profile 2/2 SER-Saddle- Mean per Object : Mean

Nucleus Symmetry 02 SER-Edge- Mean per Object : Mean
Nucleus Symmetry 03 SER-Edge- Mean per Object : Mean
Nucleus Symmetry 04 SER-Edge- Mean per Object : Mean
Nucleus Symmetry 05 SER-Edge- Mean per Object : Mean
Nucleus Symmetry 12 SER-Edge- Mean per Object : Mean
Nucleus Symmetry 13 SER-Edge- Mean per Object : Mean
Nucleus Symmetry 14 SER-Edge- Mean per Object : Mean
Nucleus Symmetry 15 SER-Edge- Mean per Object : Mean
Nucleus Threshold Compactness 30% SER-Edge- Mean per Object : Mean
Nucleus Threshold Compactness 40% SER-Edge- Mean per Object : Mean
Nucleus Threshold Compactness 50% SER-Edge- Mean per Object : Mean
Nucleus Threshold Compactness 60% SER-Edge- Mean per Object : Mean
Nucleus Axial Small Length SER-Edge- Mean per Object : Mean
Nucleus Axial Length Ratio SER-Edge- Mean per Object : Mean
Nucleus Radial Mean SER-Edge- Mean per Object : Mean
Nucleus Radial Relative Deviation SER-Edge- Mean per Object : Mean
Nucleus Radial Mean Ratio SER-Edge- Mean per Object : Mean
Nucleus Profile 1/2 SER-Edge- Mean per Object : Mean
Nucleus Profile 2/2 SER-Edge- Mean per Object : Mean
Nucleus Haralick Correlation 1 px- Mean per Object : Mean
Nucleus Haralick Contrast 1 px- Mean per Object : Mean
Nucleus Haralick Sum Variance 1 px- Mean per Object : Mean
Nucleus Haralick Homogeneity 1 px- Mean per Object : Mean
Nucleus HOECHST 33342 Gabor Min 2 px w2- Mean per Object : Mean
Nucleus HOECHST 33342 Gabor Max 2 px w2- Mean per Object : Mean
Nucleus SER Spot 2 px- Mean per Object : Mean
Nucleus SER Hole 2 px- Mean per Object : Mean
Nucleus SER Edge 2 px- Mean per Object : Mean
Nucleus SER Ridge 2 px- Mean per Object : Mean
Nucleus SER Valley 2 px- Mean per Object : Mean
Nucleus SER Saddle 2 px- Mean per Object : Mean
Nucleus SER Bright 2 px- Mean per Object : Mean
Nucleus SER Dark 2 px- Mean per Object : Mean

Object Results
Population : Image Region : None
Population : spheroid : None
Population : Nuclei : None
Population : Final Nuclei : Use Selected Well Results
Population : Final spheroid : Use Selected Well Results

Acapella version: 5.0.1.124082. Timestamp: 2021-10-01 12:05:24 -0400.
