# Supplementary material for: Machine learning-assisted high-content imaging analysis of 3D MCF7 microtissues for estrogenic effect prediction
Source: Sci Rep. 2024 Feb 6;14:2999. doi: 10.1038/s41598-024-53323-6 (PMC10844358; doi:10.1038/s41598-024-53323-6)
Supplement: Supplementary file 3 — Supplementary Information 3. [file 41598_2024_53323_MOESM3_ESM.pdf]

2D slice analysis

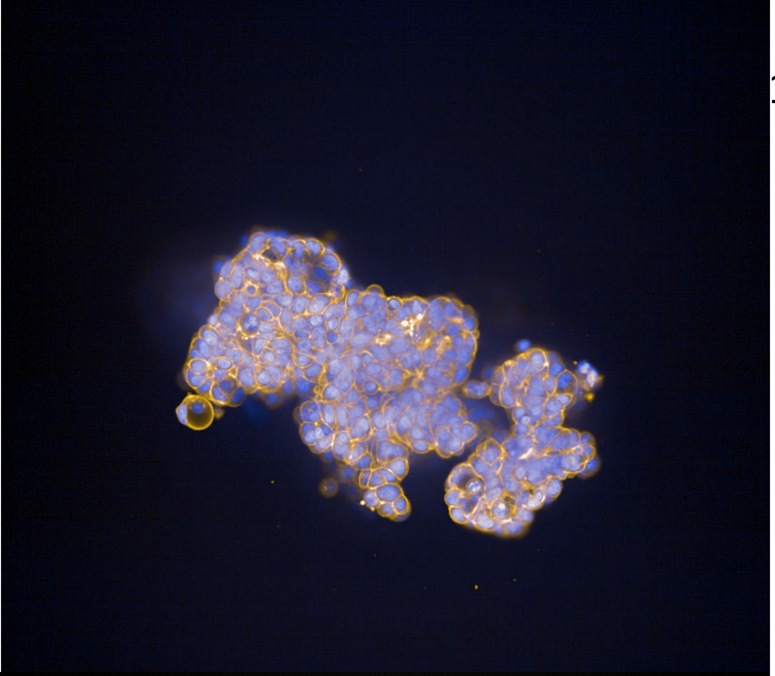

1. Input Image
   Hoechst and
   rhodamine phalloidin

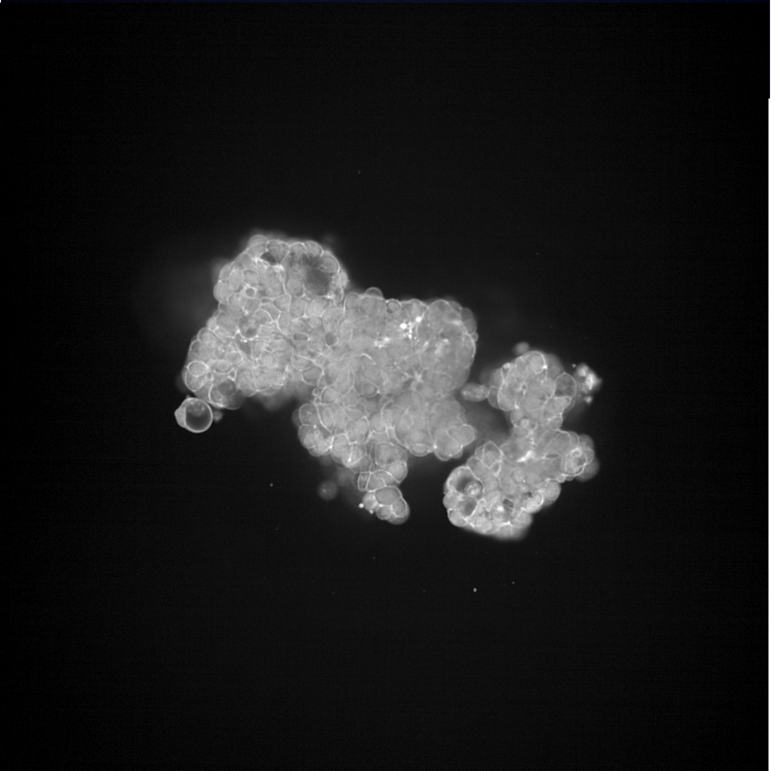

2. Calculate Image
   Combine two channels

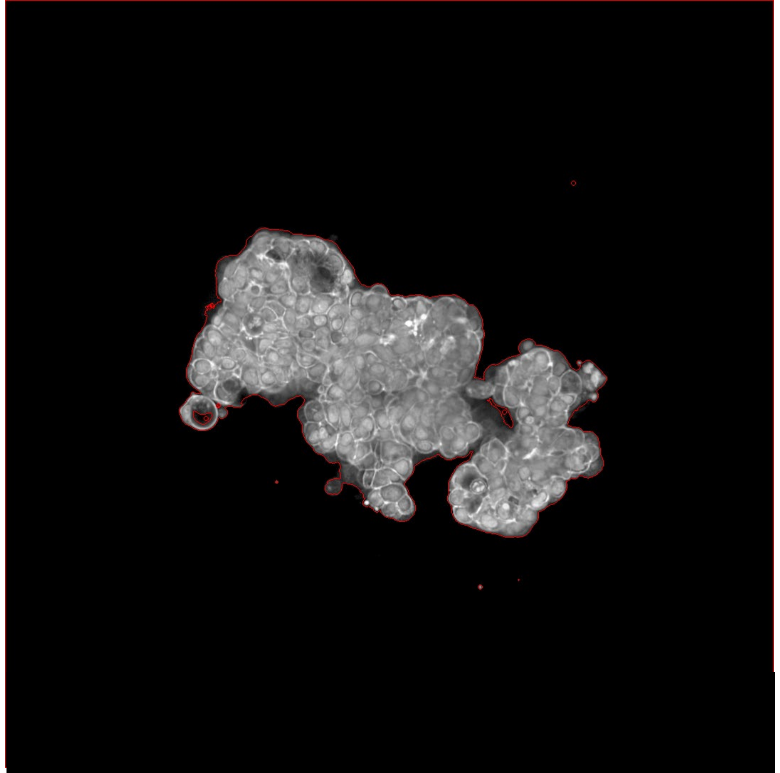

3. Calculate Image
   Subtract background

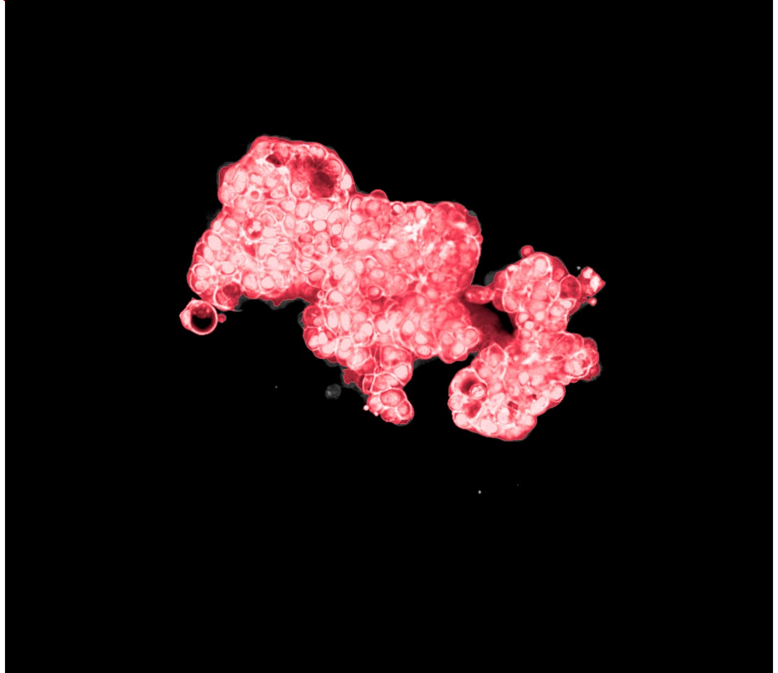

4. Find Image Region
   Absolute Threshold

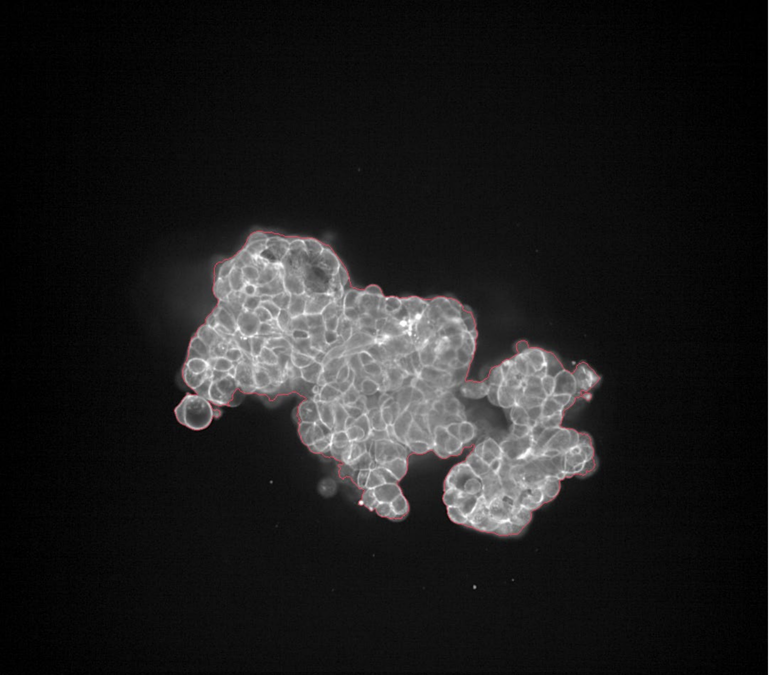

5. Calculate position properties

nearest neighbor distance

Contact area with neighbors

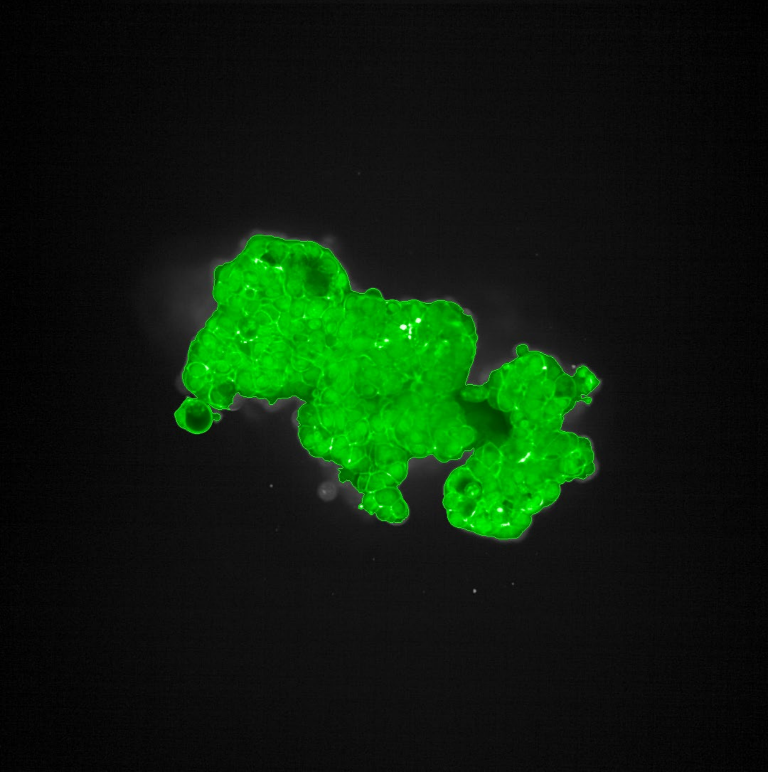

6. Select population

remove border objects

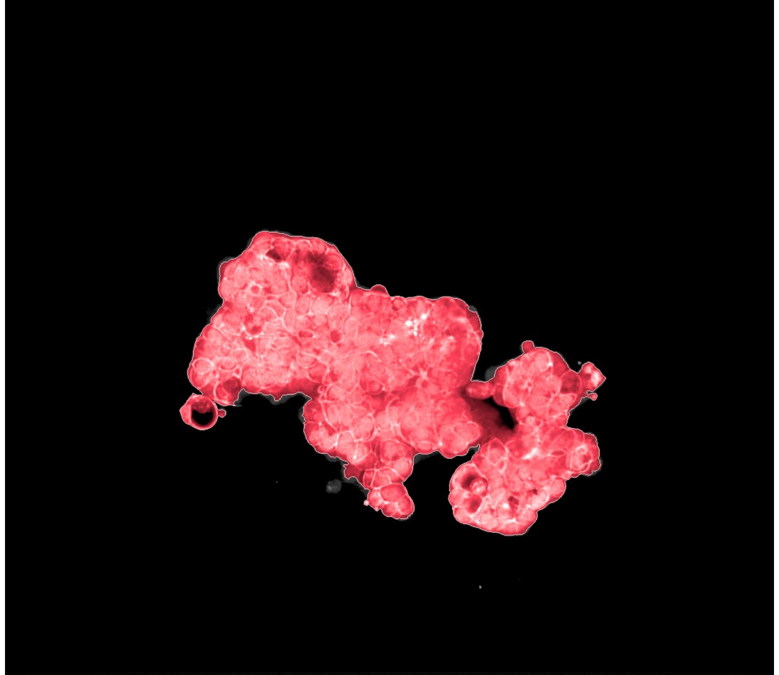

7 Find Image Region

select whole tissue

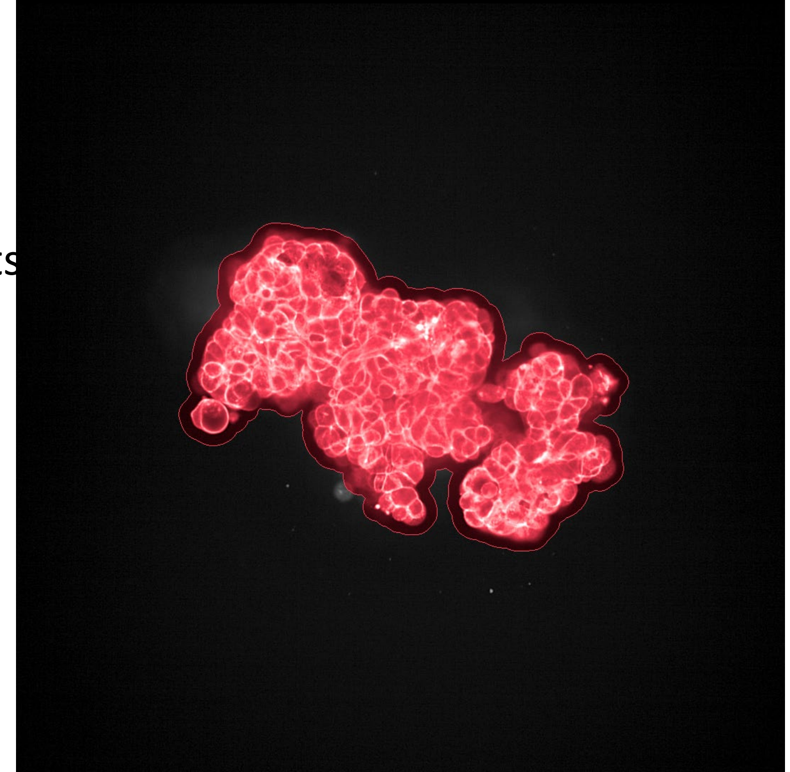

8 Select region

Enlarge border by 10 uM

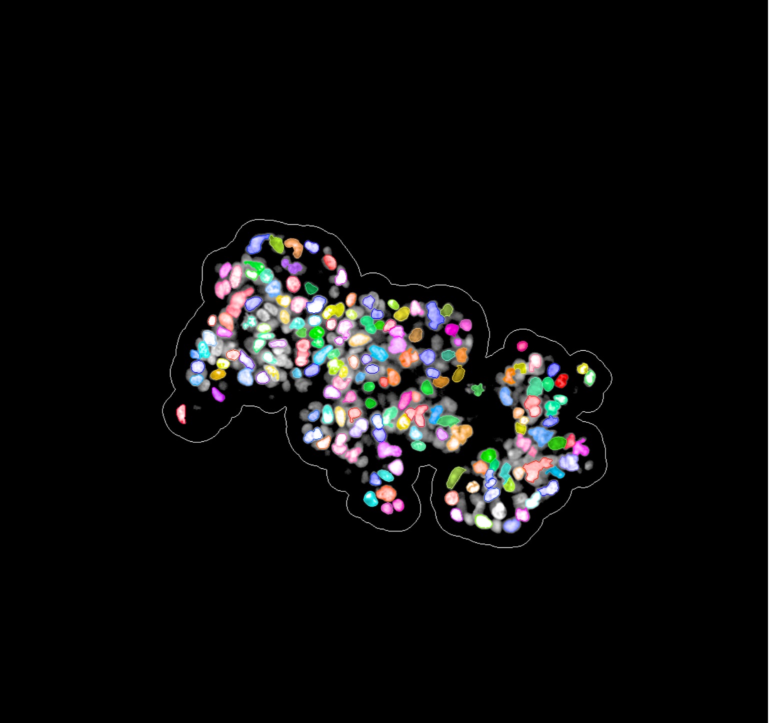

9 Find Nuclei

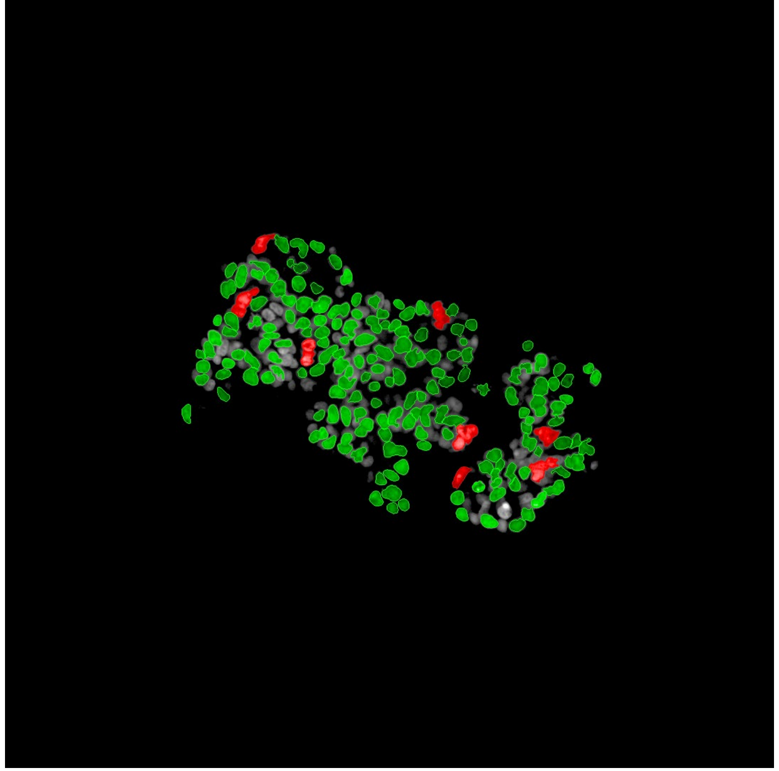

11 select population area and length cut off

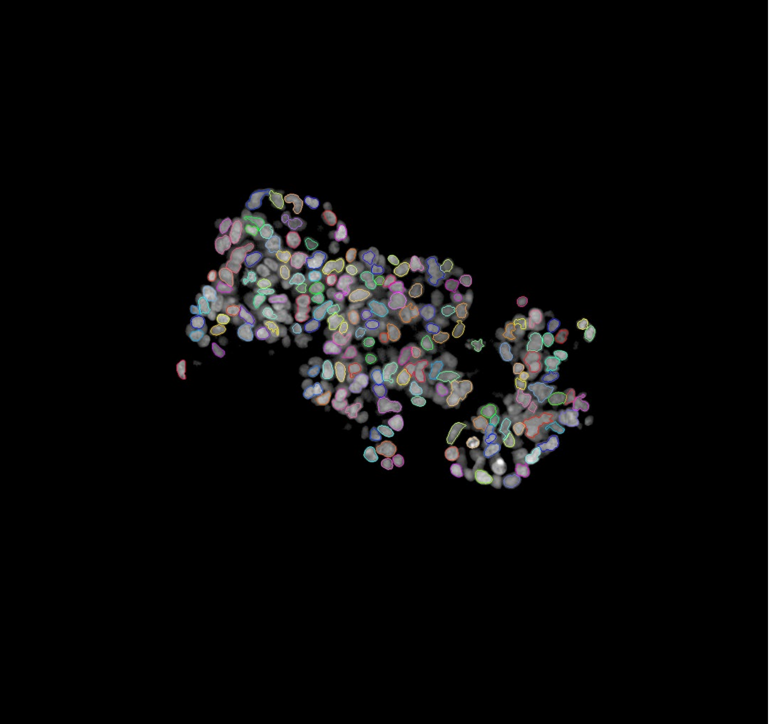

10 Calculate morphology properties area, width, length of nuclei

- Calculate Intensity Property: Alexa 568
- Calculate Intensity Property: Hoescht
- Calculate Intensity Property: Nuclei Hoescht
- Calculate Morphology Properties: Final spheroid (all selected)
- Calculate Morphology Properties: Final spheroid (STAR Alexa channel, sliding parabola and Texture SER)
- Calculate Texture Properties: Final spheroid (Alexa 568 SER features)
- Calculate Texture Properties: Final spheroid (Alexa 568 Haralick features)
- Calculate Texture Properties: Final spheroid (Alexa 568 Gabor features)
- Calculate Morphology Properties: Final Nuclei (all selected)
- Calculate Morphology Properties: Final Nuclei (STAR Hoescht channel, sliding parabola and Texture SER)
- Calculate Texture Properties: Final Nuclei (hoescht, Haralick features)
- Calculate Texture Properties: Final Nuclei (Hoechst, Gabor features)
- Calculate Texture Properties: Final Nuclei (Hoechst SER features)
- Calculate Properties: Population :Final spheroid and related population: Final nuclei
- Results: List of Outputs and object results
