# Supplementary material for: Machine learning-assisted high-content imaging analysis of 3D MCF7 microtissues for estrogenic effect prediction
Source: Sci Rep. 2024 Feb 6;14:2999. doi: 10.1038/s41598-024-53323-6 (PMC10844358; doi:10.1038/s41598-024-53323-6)

PPT Data Normalization Method Selection _Original

Data: E2 vs. DCC, 6 Groups

Feature selection method: OPLS-DA

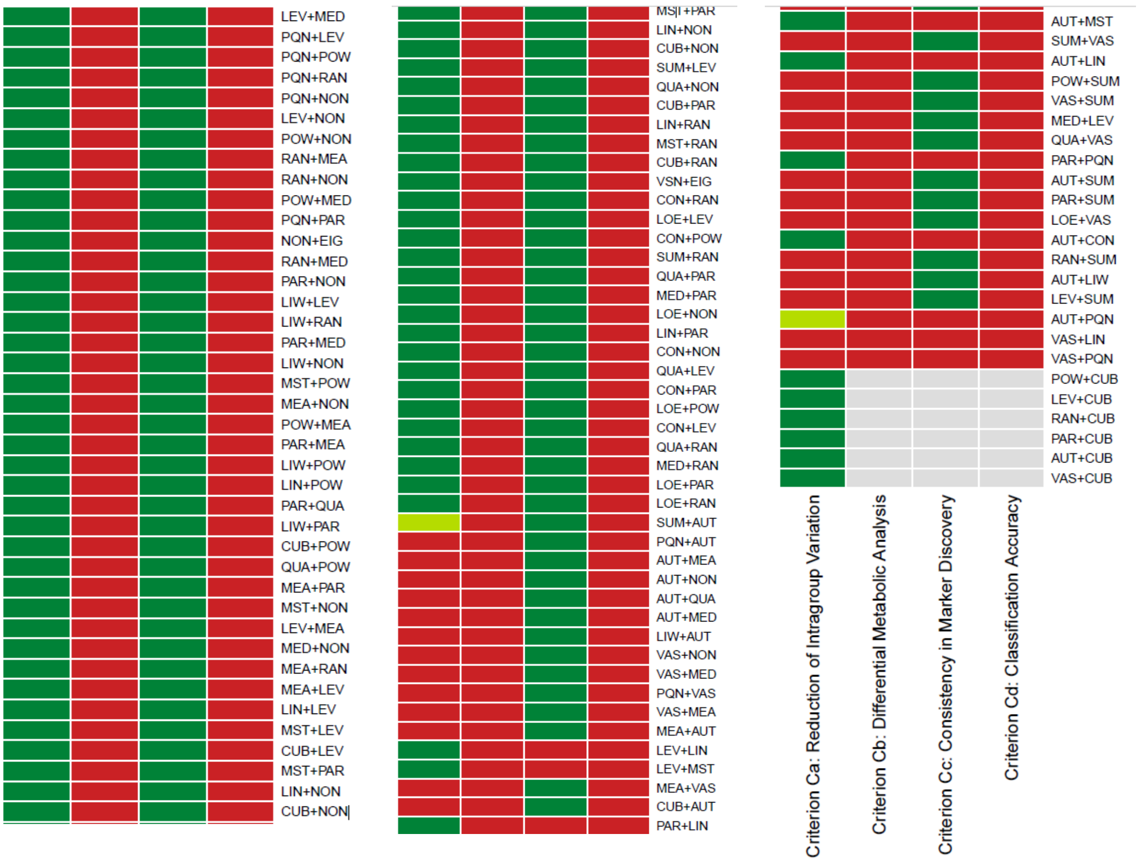

PPT Data Normalization Method Selection _Original
Data: E2 vs. DCC, 6 Groups
Feature selection method: ReliefF

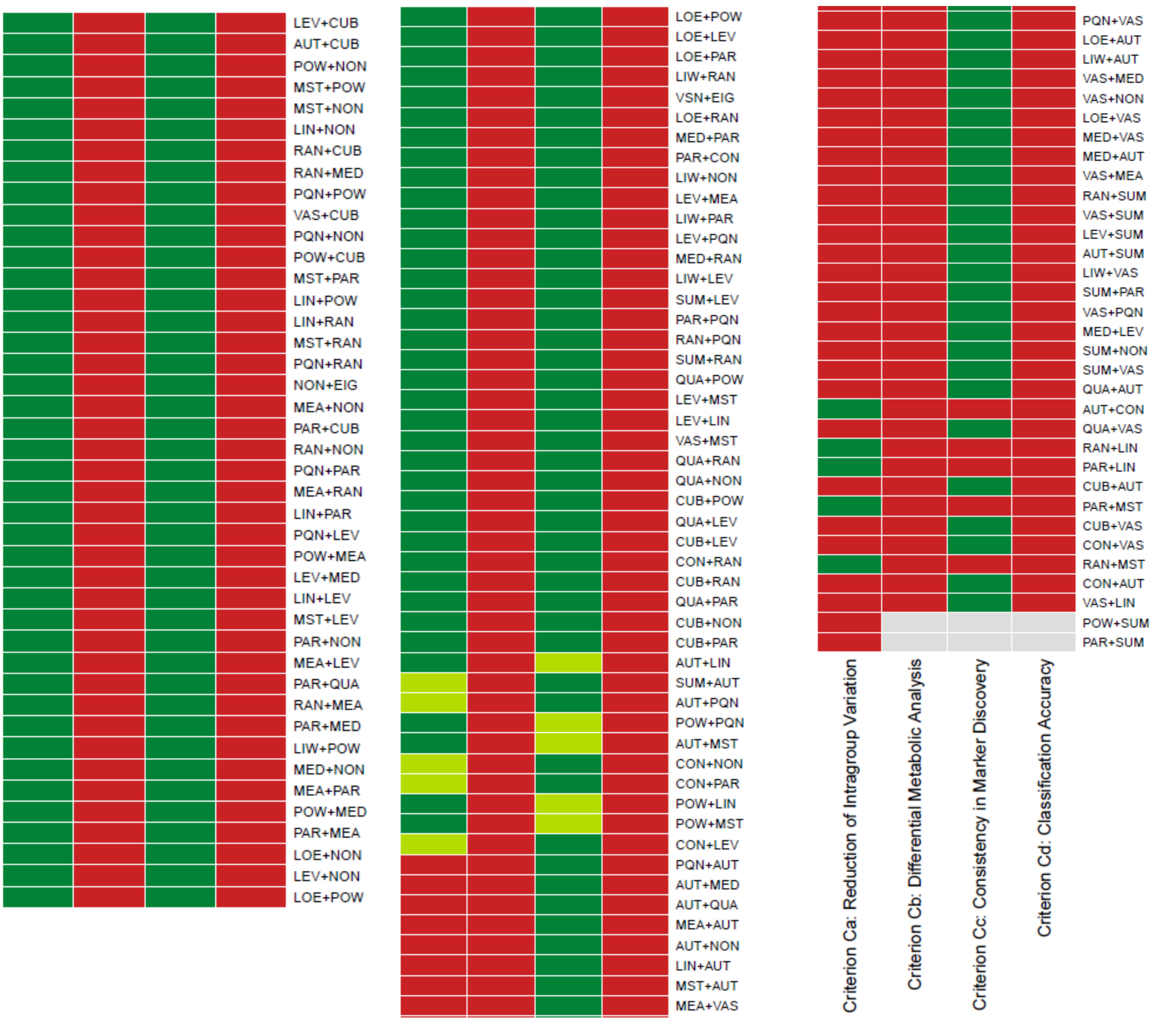

PPT Data Normalization Method Selection _Regrouped

Data: E2 vs. DCC, 4 Groups

Feature selection method: OPLS-DA

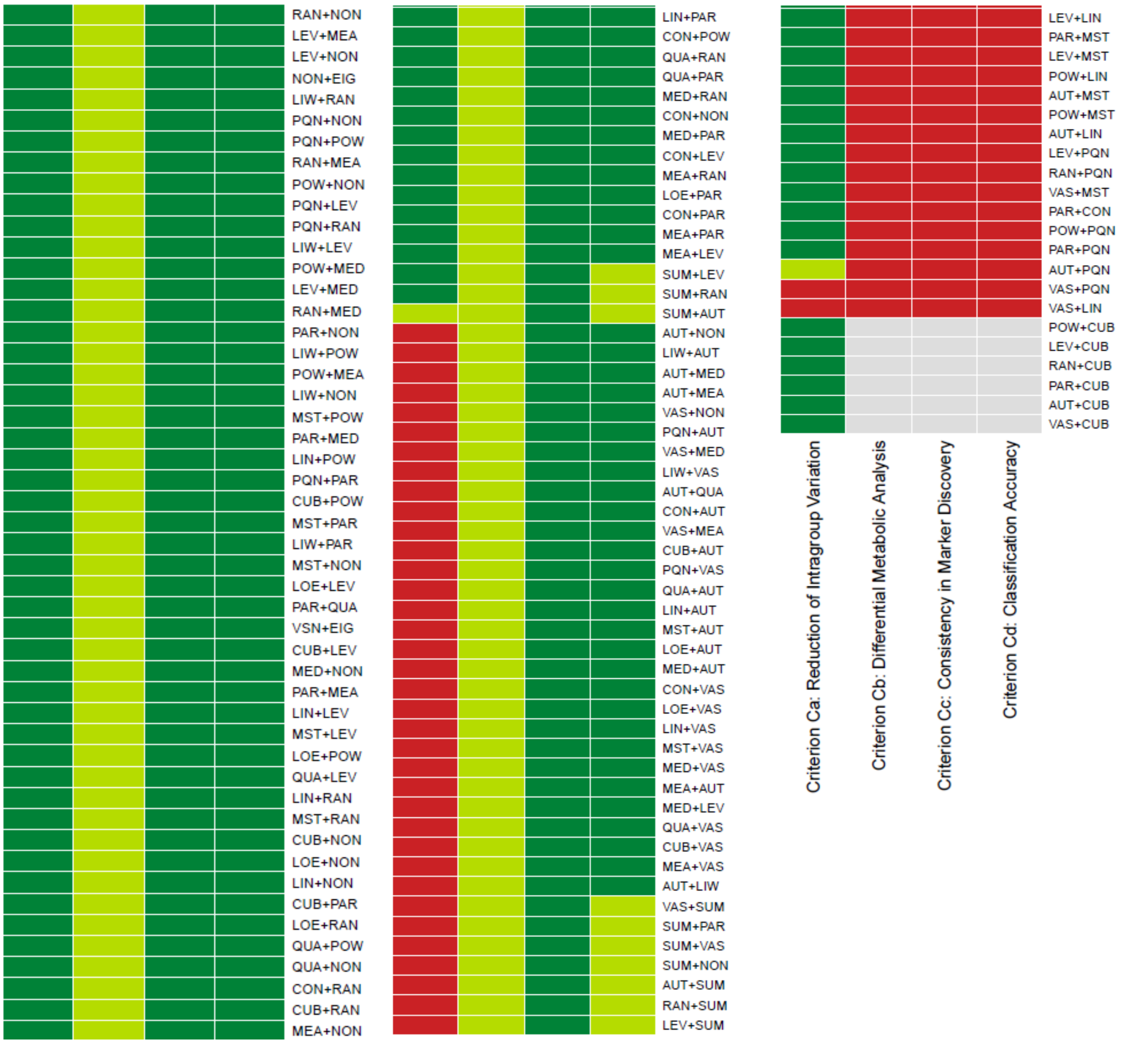

PPT Data Normalization Method Selection _Regrouped

Data: E2 vs. DCC, 4 Groups

Feature selection method: ReliefF

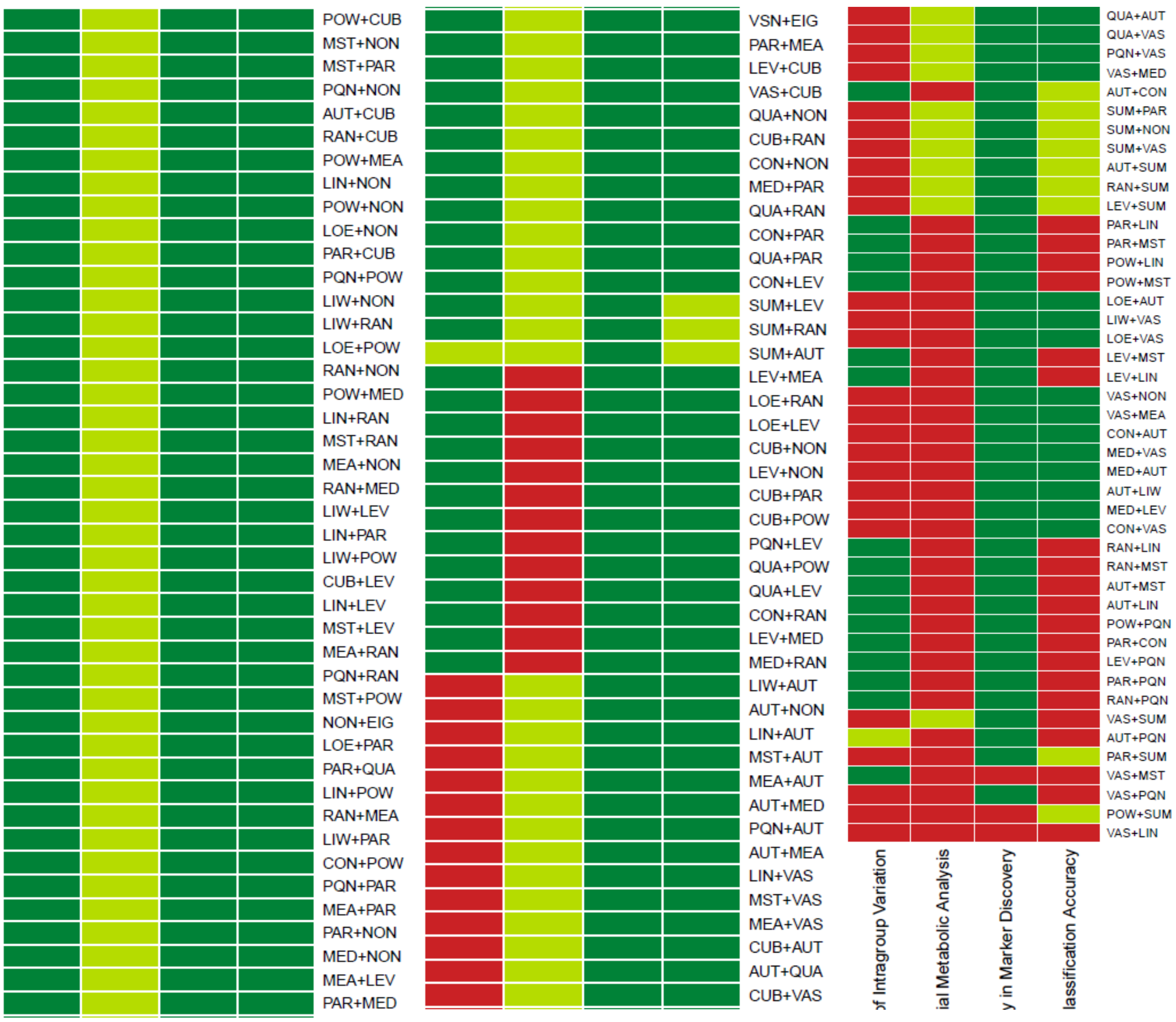

E2 Data Normalization Method Selection _Original

Data: E2 vs. DCC, 6 Groups

Feature selection method: OPLS-DA

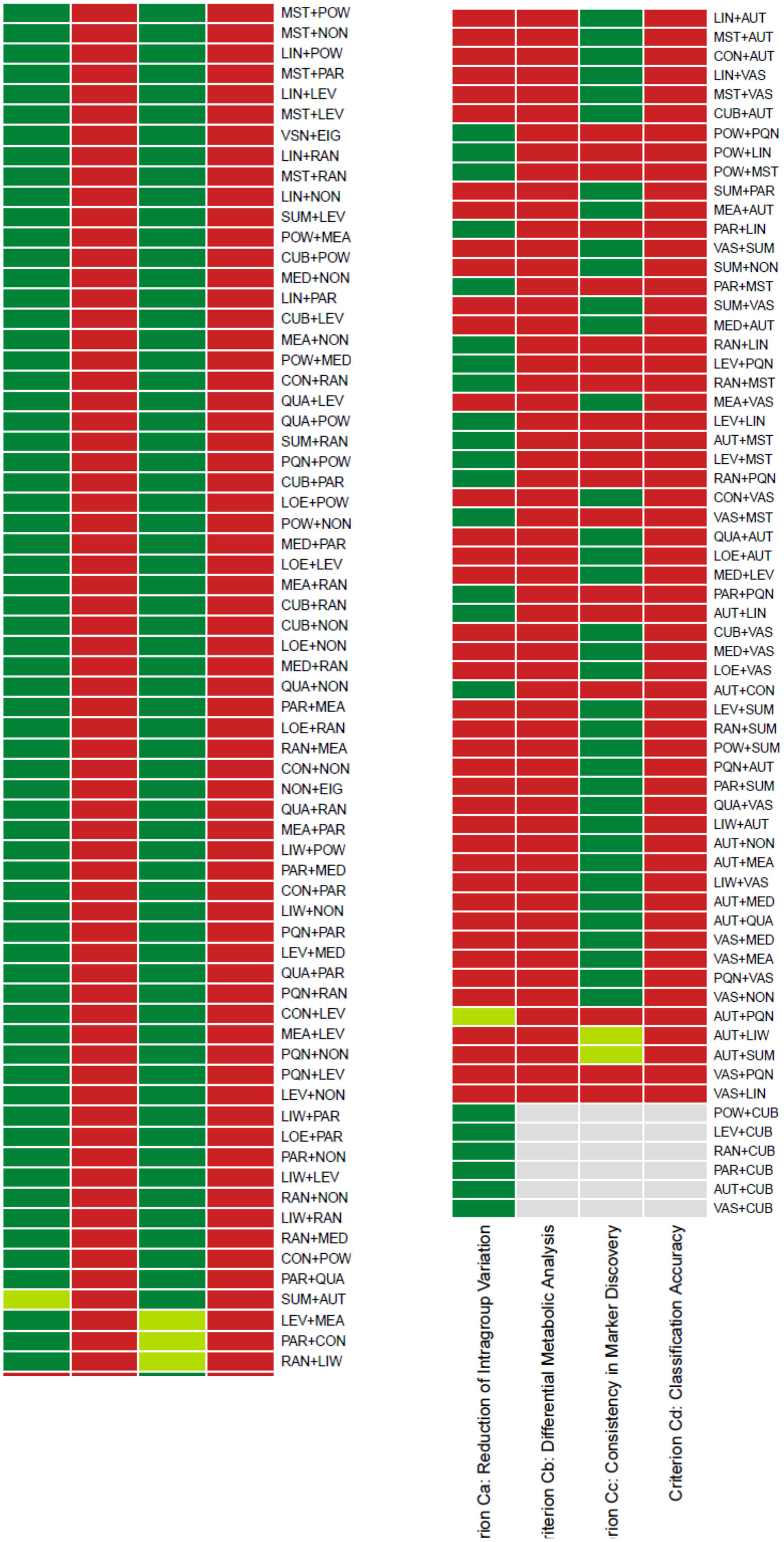

E2 Data Normalization Method Selection _Regrouped

Data: E2 vs. DCC, 4 Groups

Feature selection method: OPLS-DA

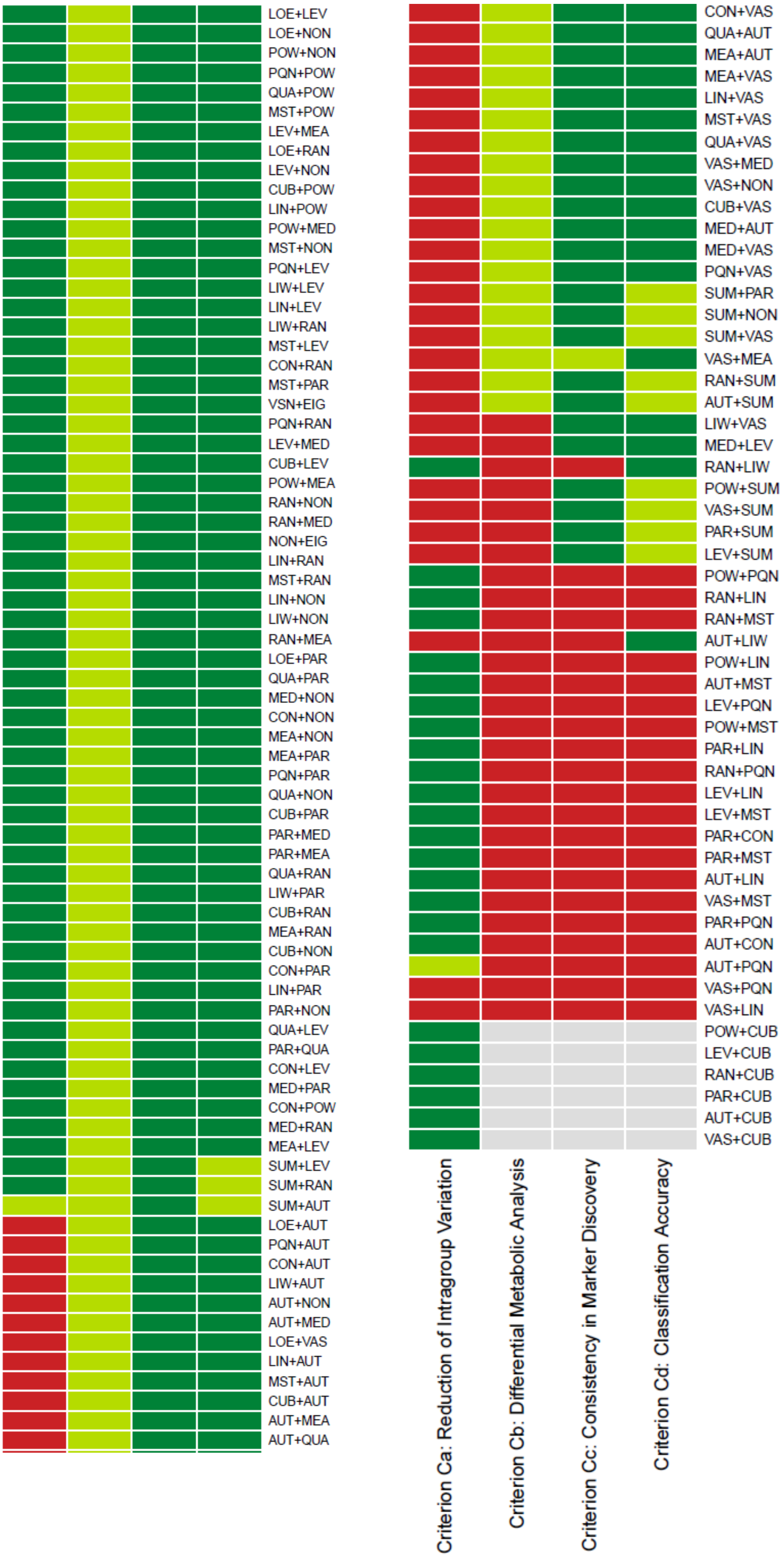

Supplement: Supplementary file 5 — Supplementary Information 5. [file 41598_2024_53323_MOESM5_ESM.pdf]
