# Supplementary figures and images for: Machine learning-assisted high-content imaging analysis of 3D MCF7 microtissues for estrogenic effect prediction
Source: Sci Rep. 2024 Feb 6;14:2999. doi: 10.1038/s41598-024-53323-6 (PMC10844358; doi:10.1038/s41598-024-53323-6)

## Slide 1
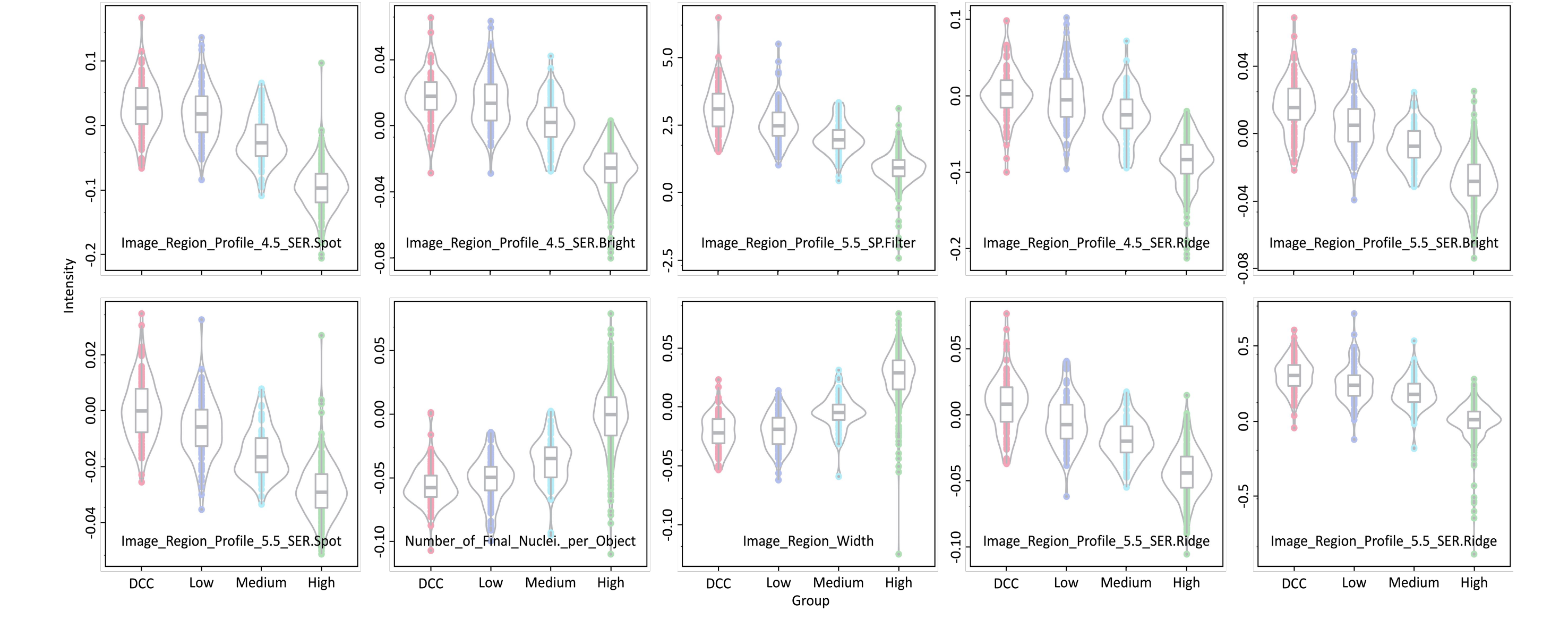

## Slide 2
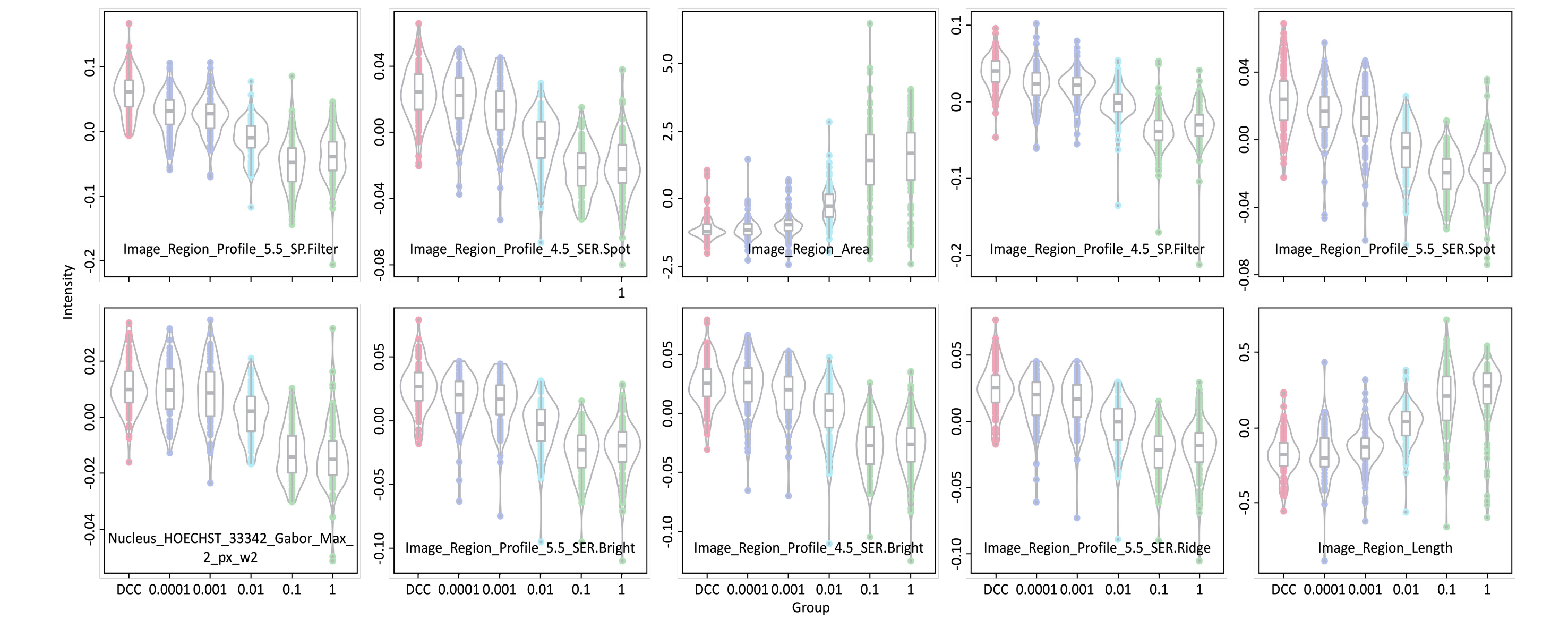

Supplement: Supplementary file 8 — Supplementary Information 8. [file 41598_2024_53323_MOESM8_ESM.pptx]

## Slide 1
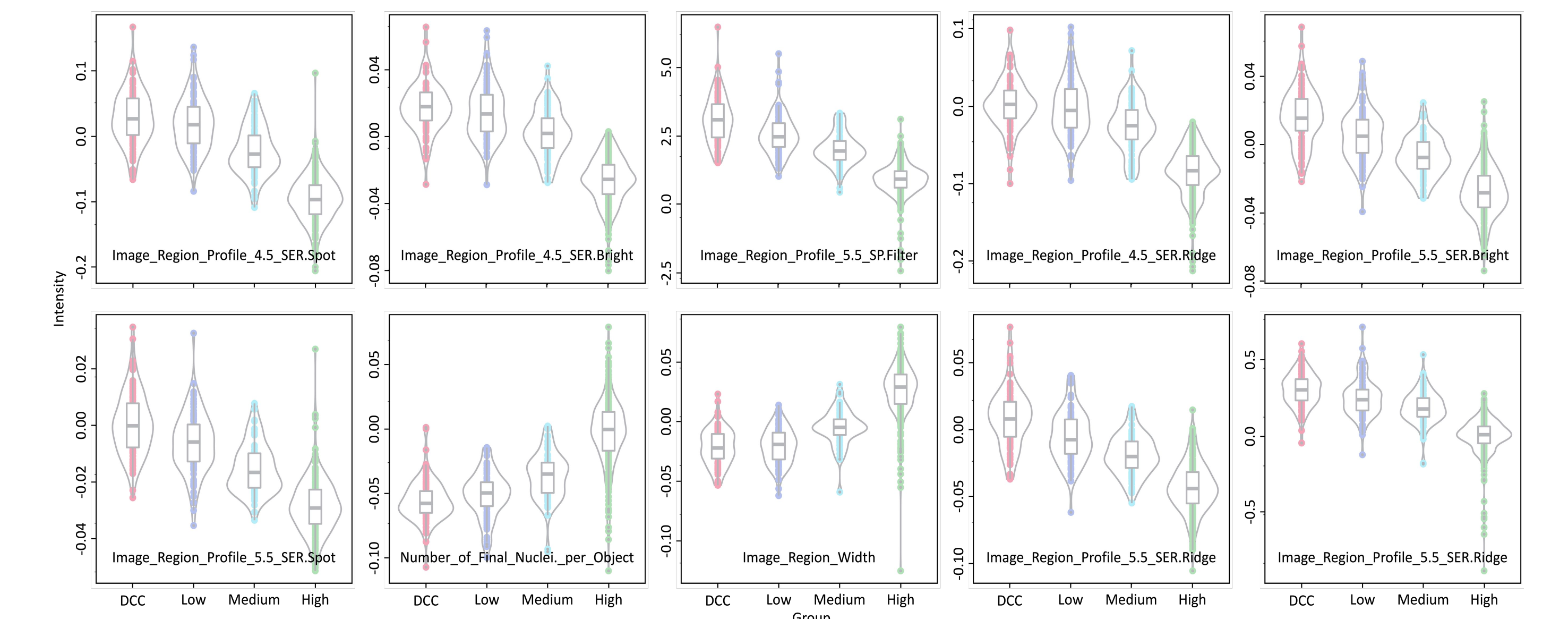

## Slide 2
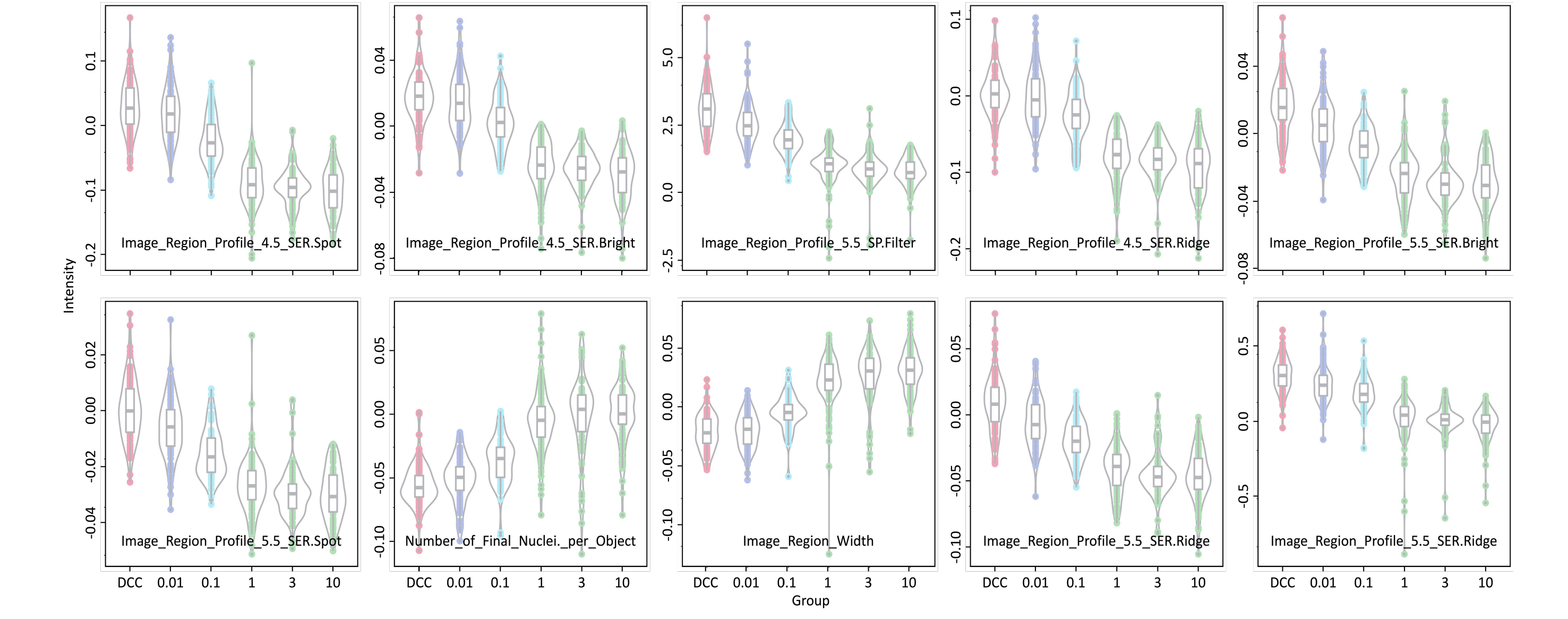

Supplement: Supplementary file 9 — Supplementary Information 9. [file 41598_2024_53323_MOESM9_ESM.pptx]
